# Identification of immune infiltration-related genes as prognostic indicators for hepatocellular carcinoma

**DOI:** 10.1186/s12885-022-09587-0

**Published:** 2022-05-05

**Authors:** Kunfu Dai, Chao Liu, Ge Guan, Jinzhen Cai, Liqun Wu

**Affiliations:** Liver Disease Center, The Affiliated Hospital of Qingdao University, No. 59 Haier Road, Qingdao, 266003 China

**Keywords:** Immune infiltration, Hepatocellular carcinoma, Bioinformatics, Prognosis, Tumour microenvironment

## Abstract

Hepatocellular carcinoma (HCC) has a high degree of malignancy and a poor prognosis. Immune infiltration-related genes have shown good predictive value in the prognosis of many solid tumours. In this study, we established and verified prognostic biomarkers consisting of immune infiltration-related genes in HCC. Gene expression data and clinical data were downloaded from The Cancer Genome Atlas (TCGA) database. Differential gene expression analysis, univariate Cox regression analysis and the least absolute shrinkage and selection operator (LASSO) regression algorithm were used to screen prognostic immune infiltration-related genes and to construct a risk scoring model. Kaplan-Meier (KM) survival plots and receiver operating characteristic (ROC) curve analysis were used to evaluate the prognostic performance of the risk scoring model in the TCGA-HCC cohort. In addition, a nomogram model with a risk score was established, and its predictive performance was verified by ROC analysis and calibration plot analysis in the TCGA-HCC cohort. Gene set enrichment analysis (GSEA) identified pathways and biological processes that may be enriched in the high-risk group. Finally, immune infiltration analysis was used to explore the characteristics of the tumour microenvironment related to the risk score. We identified 17 immune infiltration-related genes with prognostic value and constructed a risk scoring model. ROC analysis showed that the risk scoring model can accurately predict the 1-year, 3-year, and 5-year overall survival (OS) of HCC patients in the TCGA-HCC cohort. KM analysis showed that the OS of the high-risk group was significantly lower than that of the low-risk group (*P* < 0.001). The nomogram model effectively predicted the OS of HCC patients in the TCGA-HCC cohort. GSEA indicated that the immune infiltration-related genes may be involved in biological processes such as amino acid and lipid metabolism, matrisome and small molecule transportation, immune system regulation, and hepatitis virus infection. Immune infiltration analysis showed that the level of immune cell infiltration in the high-risk group was low, and the risk score was negatively correlated with infiltrating immune cells. Our prognostic model based on immune infiltration-related genes in HCC could help the prognostic assessment of HCC patients and provide potential targets for HCC inhibition.

**Supplementary Information:**

The online version contains supplementary material available at 10.1186/s12885-022-09587-0.

## Introduction

Hepatocellular carcinoma (HCC) is the most common primary liver cancer [1]. It usually develops in the context of chronic liver disease and has a poor prognosis [2]. As HCC is not sensitive to radiotherapy and chemotherapy, HCCs that cannot be radically removed lack effective treatment methods [3]. The case fatality rate is second in the world, and the five-year survival rate is less than 15% [4]. In recent years, the incidence of liver cancer has continued to rise, and it is currently the sixth most common cancer in the world [5].Immune infiltration is an important part of the tumour immune microenvironment, and it has become a hot spot in tumour research in recent years [6]. Immune infiltration-related genes refer to the genes involved in the biological process of immune infiltration [7]. The expression of immune infiltration-related genes is closely related to the occurrence and development of tumours. Many studies have confirmed the role of immune infiltration-related genes in solid tumours [8, 9]. However, the prognostic value of immune infiltration-related genes in HCC still needs to be further studied.

This study conducted a comprehensive analysis of immune infiltration-related genes in HCC. Immune infiltration-related genes were downloaded from the CIBERSORTX (https://cibersortx.stanford.edu) database. The gene expression data and clinical data of 374 HCC samples and 50 control samples were obtained from The Cancer Genome Atlas (TCGA) database. The immune infiltration-related gene expression validation data sets GSE25097, GSE87630 and GSE89377 were obtained from the Gene Expression Omnibus (GEO) database. Based on the above data resources, we conducted a comprehensive bioinformatics analysis. By identifying genes related to immune infiltration, we constructed an HCC risk scoring system and verified it in the TCGA data set. In addition, functional analysis and gene set enrichment analysis (GSEA) of immune infiltration-related genes were performed to explore the potential functions and mechanisms of these genes in HCC. Our results indicated that the signature of 17 immune infiltration-related genes could be used as an independent predictor of overall survival (OS) in HCC patients.

## Materials and methods

### Acquisition of immune infiltration-related genes

The immune infiltration-related gene data were downloaded from the CIBERSORTX database. The data provided a set of gene expression characteristics of 22 immune cell subtypes (LM22) [10]. The list of immune infiltration-related genes is shown in Table S1.

### Data set acquisition and data processing

The gene expression data and clinical data of 374 HCC samples and 50 control samples were obtained from the TCGA database. The immune infiltration-related gene expression validation data sets GSE25097, GSE87630 and GSE89377 were obtained from the GEO database. The DESeq2 algorithm was used for gene expression data processing [11]. HCC patients without prognostic information were excluded from the prognostic analysis of this study. As the data resources involved in this study were all obtained from online databases, ethics committee approval was not required.

### Differentially expressed gene (DEG) screening and identification of immune infiltration-related genes

First, we used the “DESeq2” package to analyse the DEGs between TCGA-HCC samples and normal liver samples. An adj*P* value < 0.05 and |log_2_-fold change| > 1 were used to screen DEGs. The DEGs obtained in the above steps and 636 immune infiltration-related genes were analysed by Venn diagram. A total of 89 immune infiltration-related genes were identified for downstream analysis. The gene expression matrices of the GSE25097, GSE87630 and GSE89377 data sets were downloaded from the GEO database. The gene expression heatmap of the 89 immune infiltration-related genes was drawn by the “ComplexHeatmap” package for R software (version 3.6.3). Functional enrichment analysis and visualization of 89 immune infiltration-related genes were performed by the “clusterProfiler”, “org.Hs.eg.db”, and “GOplot” packages [12, 13].

### Construction and verification of the risk scoring system

First, univariate Cox regression analysis was performed on the 89 immune infiltration-related genes. A total of 27 immune infiltration-related genes with a *P* value< 0.05 were selected for subsequent analysis. Least absolute shrinkage and selection operator (LASSO) tenfold cross-validation was performed on the 27 immune infiltration-related genes by using the “glmnet” and “survival” packages. The 17 most valuable predictive genes and risk score models were obtained through the above analysis. Subsequently, the 17 obtained genes were integrated into risk characteristics, and the risk scoring system was established based on the standardized gene expression values and their coefficients. The risk scoring system was established based on the following formula: Risk score = ∑ ${}_{\mathrm{i}=1}^{\mathrm{n}}$ expr_genei_ × coefficient_genei_ [14]. Through the “edgeR” package, the TMM algorithm was used to calculate the normalized gene expression levels. A risk factor plot was drawn by the “ggplot2” package. The “timeROC” package was used to draw receiver operating characteristic (ROC) curves. According to the median risk score, the patients were divided into a high-risk group and a low-risk group. The “survminer” package was used to draw survival curves. Dot plots were drawn using the “ggplot2” software package to determine the link between the risk score and clinical characteristics.

### Construction and evaluation of the nomogram

To evaluate whether the risk scoring system can be used as an independent predictor, univariate and multivariate Cox regression analyses were performed on each clinicopathological parameter, including histologic grade, T stage, residual tumour, pathologic stage, vascular invasion, and alpha-fetoprotein (AFP). All independent prognostic parameters were used to construct a nomogram using the “rms” package to predict OS probabilities at 1, 3, and 5 years. The discriminative ability of the nomogram was verified by ROC and calibration analyses.

### GSEA

The above R software packages were used to identify the DEGs between the high-risk group and the low-risk group in the TCGA data set. The “clusterProfiler” package was used for GSEA. The “ggplot2” package was used for visualization.

### Immune cell infiltration level analysis

The “GSVA” package was used to analyse the level of immune cell infiltration between the high-risk group and the low-risk group [15, 16].

### Statistical analysis

All statistical analyses in this study were performed by R software (version 3.6.3). The log-rank test was used for Kaplan-Meier survival analysis. Hazard ratios (HRs) and 95% confidence intervals (CIs) were calculated in the regression analysis. Student’s t test and the Kruskal–Wallis test were used for comparisons between groups. A two-tailed *P* value of < 0.05 was considered statistically significant.

## Results

### Identification of immune infiltration-related genes in HCC patients

**Fig. 1:**
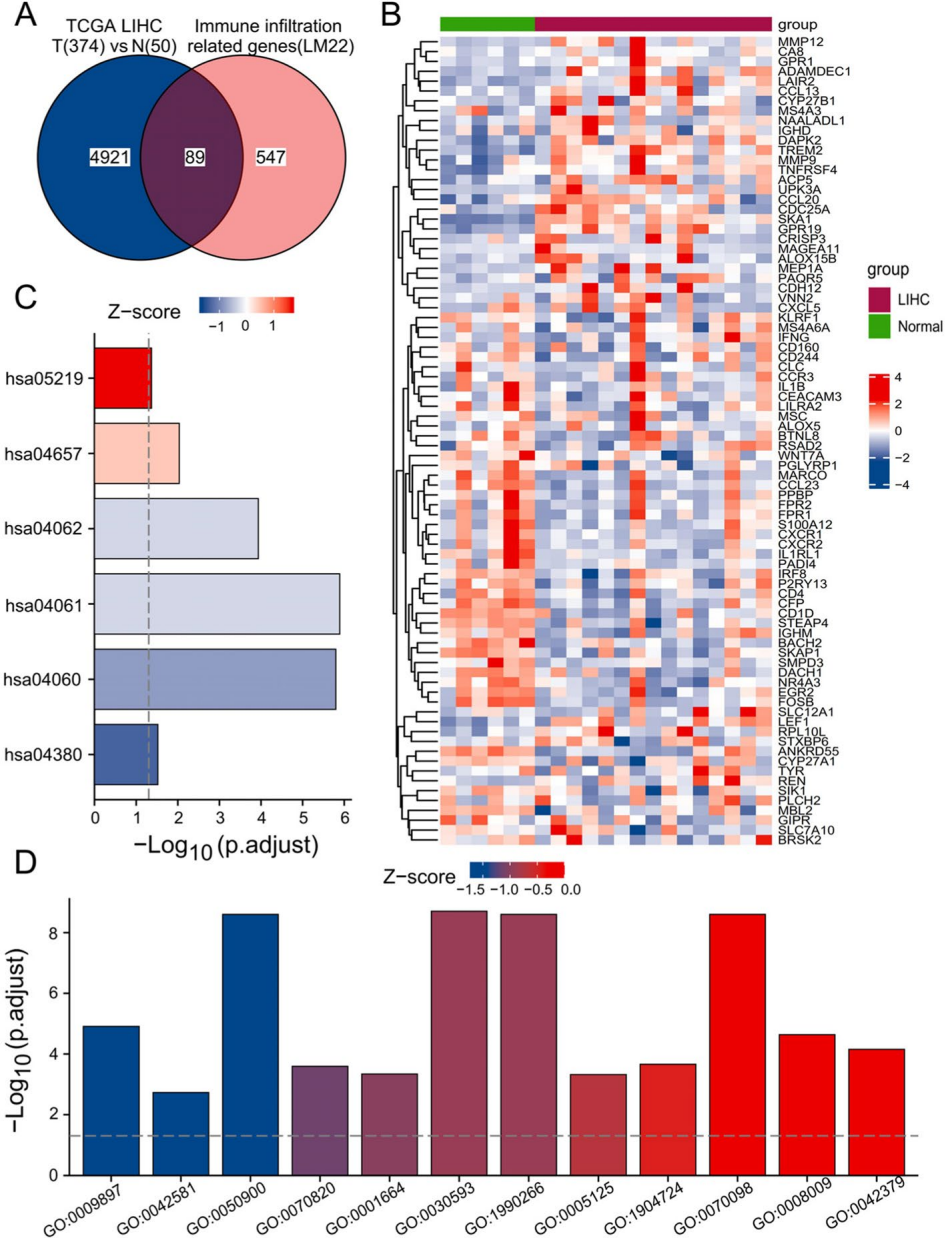
Identification and functional enrichment analysis of immune infiltration-related genes between the TCGA-HCC cohort and normal liver samples. **A** Venn diagram of the intersection between immune infiltration-related genes and DEGs identified by the DESeq2 algorithm. **B** Heat map of 89 DEGs related to immune infiltration in the data set GSE25097. Terms of Gene Ontology (GO) enrichment analysis (**C**) and KEGG pathways (**D**) related to the 89 immune infiltration-related genes

According to the criteria for DEGs, we used the DESeq2 algorithm and identified 5010 DEGs between 374 TCGA-HCC samples and 50 normal liver samples. The 5010 identified DEGs and 636 immune infiltration-related genes obtained from the CIBERSORTX database were used for Venn diagram analysis. Through the above analysis, we obtained 89 immune infiltration-related genes in HCC (Fig. 1A). Then, we verified the expression of the 89 immune infiltration-related genes in the GSE25097, GSE87630 and GSE89377 data sets from the GEO database (Fig. 1B, Fig. S1, and Fig. S2). We conducted further enrichment analysis to explore the functions of the selected genes. The genes were significantly enriched in neutrophil chemotaxis, neutrophil migration, the external side of the plasma membrane, tertiary granule lumen, chemokine activity, and chemokine receptor binding (Fig. 1C). Kyoto Encyclopedia of Genes and Genomes (KEGG) enrichment analysis showed that viral protein interaction with cytokine and cytokine receptor, cytokine-cytokine receptor interaction, and chemokine signalling pathway were all significantly enriched (Fig. 1D). The complete results of the enrichment analysis are shown in Table S2.

### Construction and assessment of the risk scoring system

**Fig. 2:**
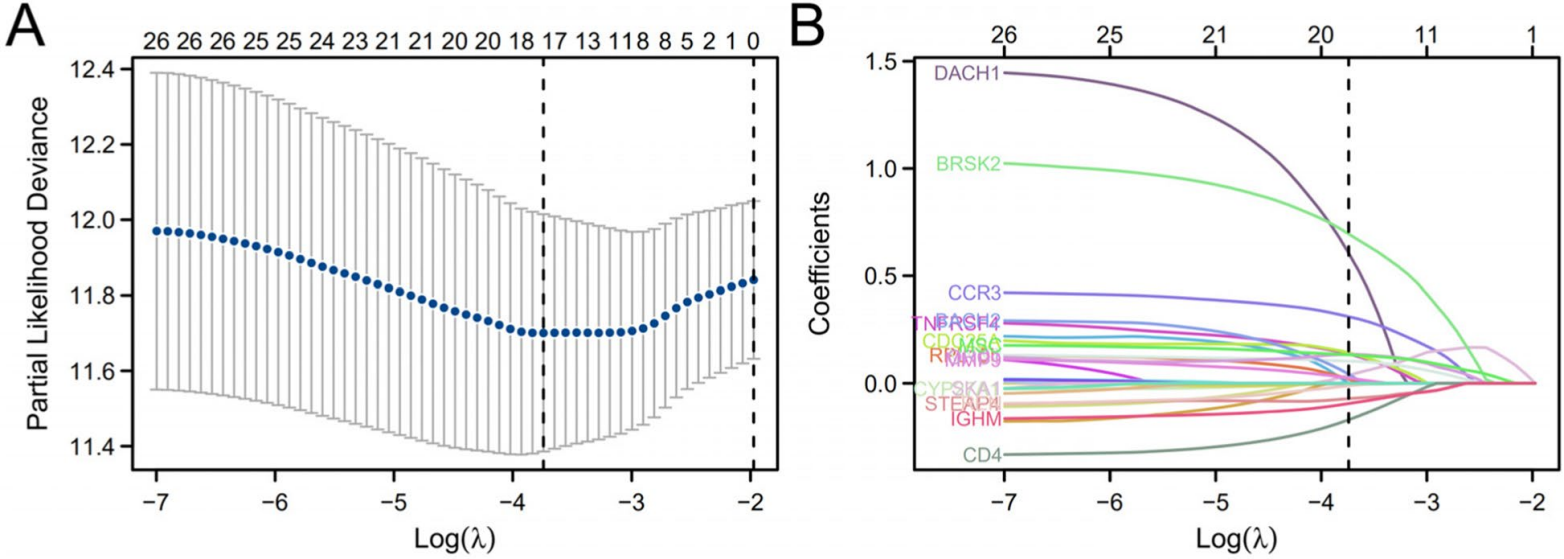
Demonstration of DEGs with univariate Cox regression *P* value < 0.05. **A** The LASSO regression model of the 27 immune infiltration-related genes performed by Lasso-ten-fold cross-validation. **B** The coefficient distribution in the LASSO regression model

First, univariate Cox regression analysis was performed to explore the relationship between the expression levels of 89 immune infiltration-related genes and the OS times of patients in the TCGA-HCC cohort. Using the cut-off value of Cox *P* < 0.05, 27 potential predictive genes related to OS were screened out (Table S3). Then, LASSO regression analysis was used to refine the gene sets (Fig. 2A, B). Seventeen genes were identified as the most valuable predictive genes, and the risk scoring system was established based on the above formula (Table 1). Kaplan–Meier analysis of the 17 genes is shown in Fig. S3.

**Table 1:** Seventeen immune infiltration-related genes identified by univariate COX regression analysis

Gene	Description	HR (95% CI)	***P*** value
ORC1	origin recognition complex, subunit 1	1.842 (1.314–2.613)	**0.001**
VNN2	vanin 2	1.862 (1.228–2.837)	**0.003**
STEAP4	STEAP family member 4	0.459 (0.341–0.707)	**< 0.001**
SKA1	spindle and kinetochore associated complex subunit 1	2.094 (1.482–2.964)	**< 0.001**
BRSK2	BR serine/threonine kinase 2	1.628 (1.127–2.341)	**0.009**
MSC	musculin	1.683 (1.164–2.417)	**0.006**
CCR3	chemokine (C-C motif) receptor 3	2.426 (1.687–3.491)	**< 0.001**
IGHM	immunoglobulin heavy constant mu	0.673 (0.465–0.964)	**0.029**
CYP27A1	cytochrome P450, family 27, subfamily A, polypeptide 1	0.469 (0.339–0.697)	**< 0.001**
DACH1	dachshund family transcription factor 1	1.461 (1.032–2.064)	**0.032**
TNFRSF4	tumor necrosis factor receptor superfamily, member 4	1.788 (1.264–2.537)	**0.001**
RPL10L	ribosomal protein L10-like	1.762 (1.239–2.487)	**0.002**
CDC25A	cell division cycle 25A	2.096 (1.485–2.977)	**< 0.001**
REN	renin	0.639 (0.448–0.906)	**0.014**
BACH2	BTB and CNC homology 1, basic leucine zipper transcription factor 2	1.485 (1.053–2.114)	**0.024**
MMP9	matrix metallopeptidase 9	1.953 (1.306–2.891)	**0.001**
CD4	CD4 molecule	0.694 (0.491–0.981)	**0.037**

**Annotation**: *HR* Hazard Ratio, *95%CI* 95% confidence interval

**Fig. 3:**
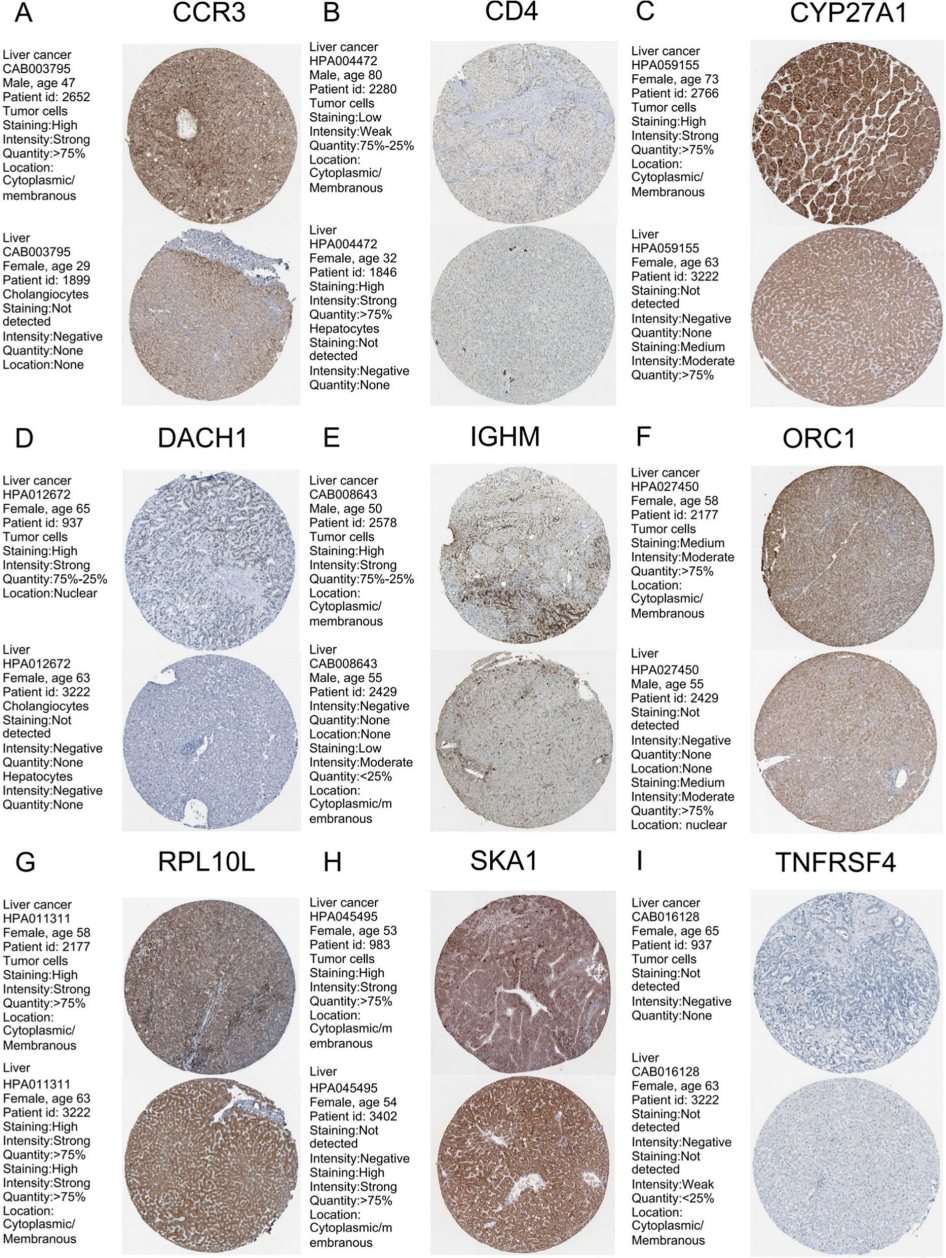
Immunohistochemical analysis of HCC and normal liver tissue determined by HPA database. **A** CCR3; **B** CD4; **C** CYP27A1; **D** DACH1; **E** IGHM; **F** ORC1; **G** RPL10L; **H** SKA1; **I** TNFRSF4

To observe the expression of these genes in HCC and normal liver tissues, we further conducted research using immunohistochemical data from the Human Protein Atlas (HPA) database. The results are shown in Fig. 3. The immunohistochemical data of some genes were temporarily unavailable from the HPA database.

**Fig. 4:**
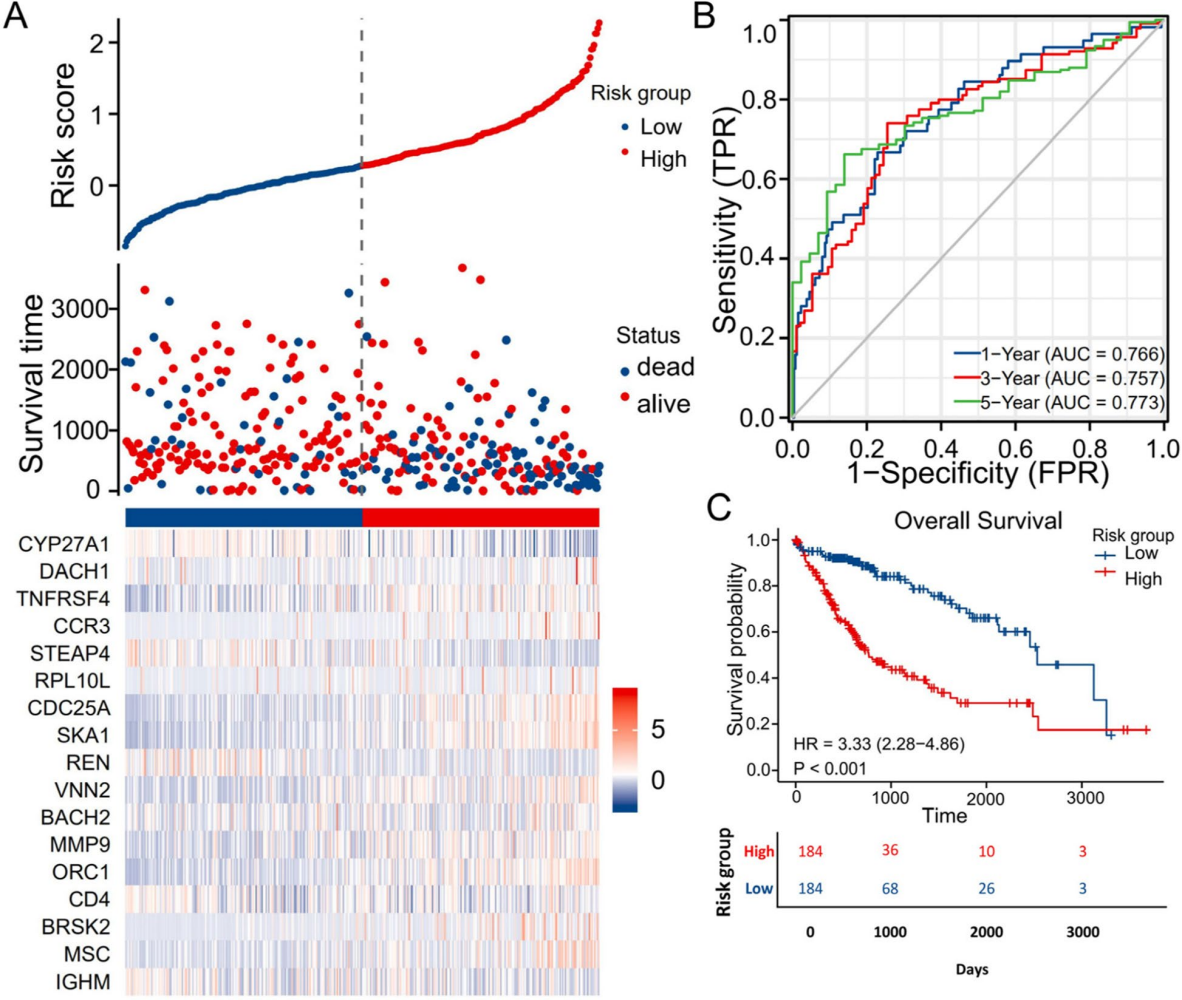
The risk score analysis, prognostic performance and survival analysis of the risk scoring model based on the differential expression of the 17 immune infiltration-related genes in TCGA-HCC patients. **A** The risk score, survival time distributions and gene expression heat map of immune infiltration-related genes in the TCGA-HCC cohort. **B** The ROC curves of the risk scoring model predicting OS of 1-year, 3-year, and 5-year in the TCGA-HCC cohort. **C** Kaplan–Meier survival analysis of the OS between the risk groups in the TCGA-HCC cohort

The risk score of each patient in the TCGA-HCC data set was calculated based on the expression levels and regression coefficients of the 17 immune infiltration-related genes. The distribution of risk scores in the TCGA-HCC data set is shown in Fig. 4A. According to the median risk score, the patients in the TCGA-HCC cohort were divided into high-risk and low-risk groups. In addition, the survival time distribution indicated that the higher the risk score was, the worse the prognosis (Fig. 4A). Figure 4A also shows the corresponding expression levels of the 17 immune infiltration-related genes. The performance of the risk scoring system according to the time ROC curves in terms of 1-year, 3-year, and 5-year prognoses is shown in Fig. 4B. The areas under the time ROC curves (AUCs) were 0.766, 0.757, and 0.773 for the 1-year, 3-year, and 5-year OS times, respectively, in the TCGA-HCC cohort. Kaplan–Meier analysis and the log-rank test showed that the prognosis of the high-risk group was significantly worse than that of the low-risk group (*P* < 0.001; Fig. 4C).

### Correlation between the risk score and clinical features

**Fig. 5:**
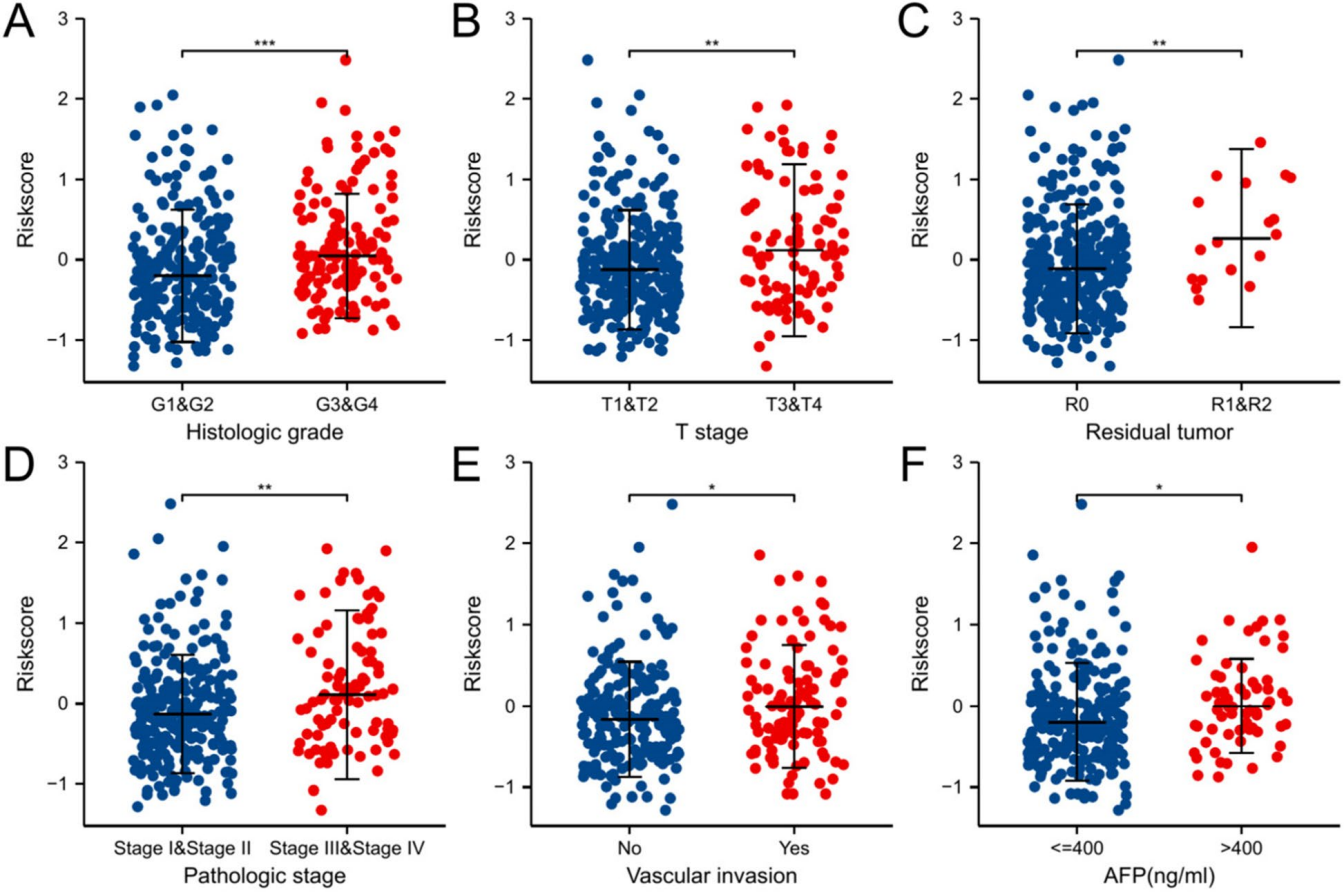
Correlation between clinical features and the immune infiltration risk score in the TCGA-HCC data set. **A** Histologic grade (G1**&**2 vs G3**&**G4), **B** T stage(T1**&**T2 vs T3**&**T4), **C** Residual tumor (R0 vs R1**&**R2), **D** Pathologic stage (Stage1**&**Stage2 vs Stage3**&**Stage4), **E** Vascular invasion (No vs Yes), **F** AFP(≤400 vs > 400). *P < 0.05, **P < 0.01, ***P < 0.001

We also analysed the association between the risk score and the clinical features of patients in the TCGA-HCC cohort. We found significant differences between the risk score and the following clinical features (Fig. 5 A–F): histological grade (G1&2 vs. G3&G4, P < 0.001), T stage (T1&T2 vs. T3&T4, *P* < 0.01), residual tumour (R0 vs. R1&R2, *P* < 0.01), pathologic stage (stage 1 & stage 2 vs. stage 3&stage 4, *P* < 0.01), vascular invasion (no vs. yes, *P* < 0.05) and AFP (≤400 vs. > 400, *P* < 0.05).

### Construction and verification of the nomogram

**Fig. 6:**
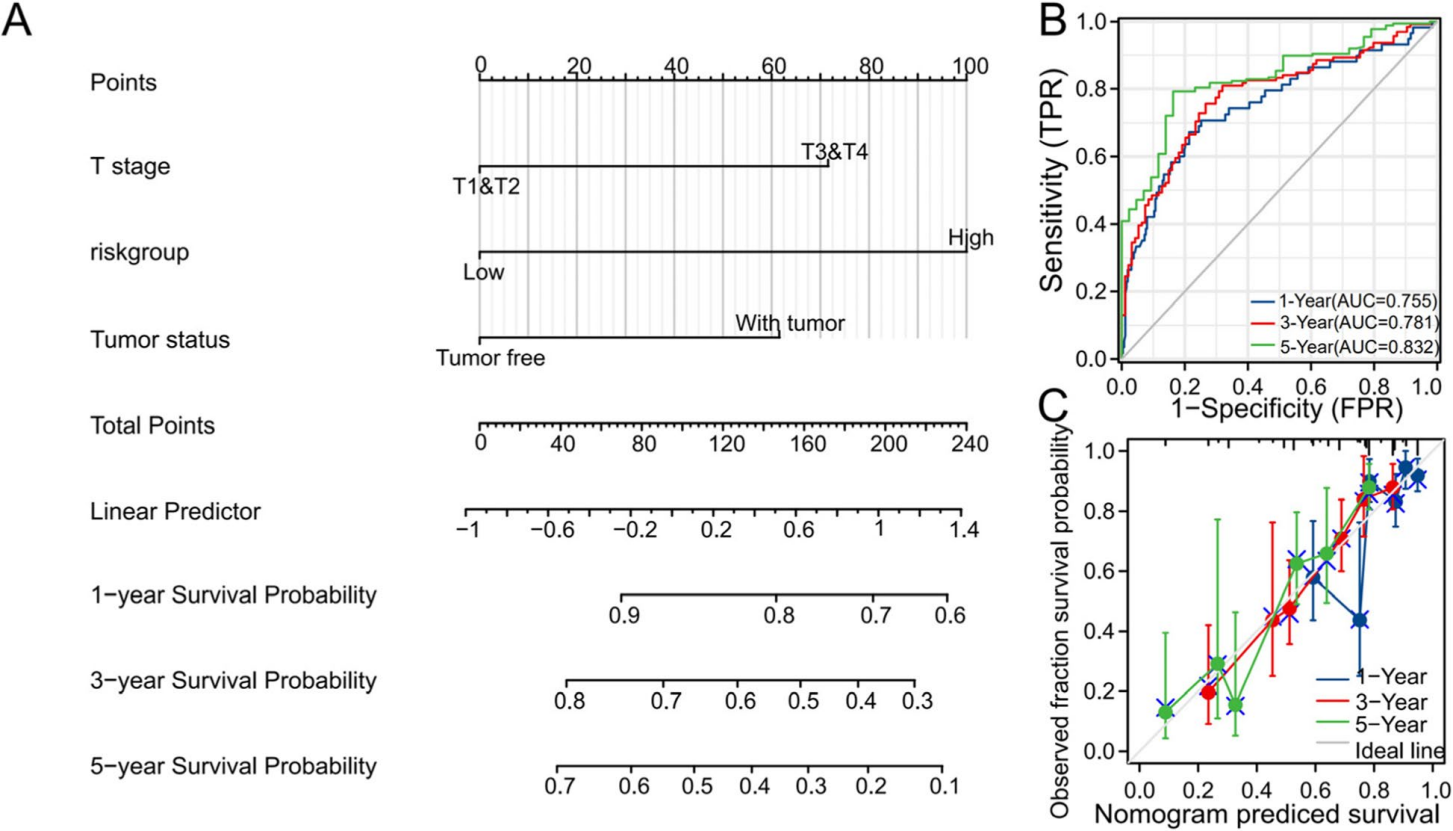
Prognostic nomogram for the 1-year, 3-year, and 5-year OS of HCC patients. **A** The independent risk factors that affect the OS of HCC patients screened by multiple Cox regression were incorporated into the nomogram model. **B** The ROC curves for predicting the nomogram of 1-year, 3-year, and 5-year OS in the TCGA-HCC cohort. **B** The nomogram calibration curves for predicting the 1-year, 3-year, and 5-year OS of the TCGA-HCC cohort

First, we performed univariate and multivariate Cox regression analyses of potential predictors, such as T stage, gender, age, residual tumour, histologic grade, AFP, vascular invasion, tumour status, and risk group, that may affect the prognosis of HCC patients (Table 2). The results showed that T stage, tumour status, and risk group were independent risk factors for OS in HCC patients. The independent predictors, including T stage, tumour status, and risk group, which affect the OS of HCC patients, were incorporated into the nomogram model (Fig. 6A). The C-index of the nomogram model we established was 0.692 (0.664–0.720). Then, we calculated the score of each HCC patient based on the nomogram and evaluated the predictive ability of the nomogram through ROC analysis. In the TCGA-HCC cohort, the nomogram AUCs for the 1-year, 3-year, and 5-year OS rates were 0.755, 0.781, and 0.832, respectively (Fig. 6B). Moreover, we used the calibration curve to evaluate the agreement of the nomogram. Compared with the ideal model, the calibration plots of the nomogram model showed good agreement for the 1-year, 3-year, and 5-year OS rates (Fig. 6C).

**Table 2:** Univariate and multivariate Cox regression analysis between the clinical features and OS in the TCGA-HCC cohort

Characteristics	Total(N)	Univariate analysis		Multivariate analysis	
		Hazard ratio (95% CI)	*P* value	Hazard ratio (95% CI)	*P* value
T stage	370				
T1&T2	277	Reference			
T4&T3	93	2.598 (1.826–3.697)	**< 0.001**	2.021 (1.389–2.941)	**< 0.001**
Gender	373				
Female	121	Reference			
Male	252	0.793 (0.557–1.130)	0.200		
Age	373				
<=60	177	Reference			
> 60	196	1.205 (0.850–1.708)	0.295		
Residual tumor	344				
R0	326	Reference			
R1&R2	18	1.604 (0.812–3.169)	0.174		
Histologic grade	368				
G1	55	Reference			
G2	178	1.162 (0.686–1.968)	0.577		
G3&G4	135	1.222 (0.710–2.103)	0.469		
AFP (ng/ml)	279				
<=400	215	Reference			
> 400	64	1.075 (0.658–1.759)	0.772		
Vascular invasion	317				
No	208	Reference			
Yes	109	1.344 (0.887–2.035)	0.163		
Tumor status	354				
Tumor free	202	Reference			
With tumor	152	2.317 (1.590–3.376)	**< 0.001**	1.832 (1.242–2.701)	**0.002**
riskgroup	373				
Low	186	Reference			
High	187	2.924 (2.014–4.244)	**< 0.001**	2.672 (1.800–3.968)	**< 0.001**

**Annotation**: *HR* Hazard Ratio, *95%CI* 95% confidence interval

### GSEA

**Fig. 7:**
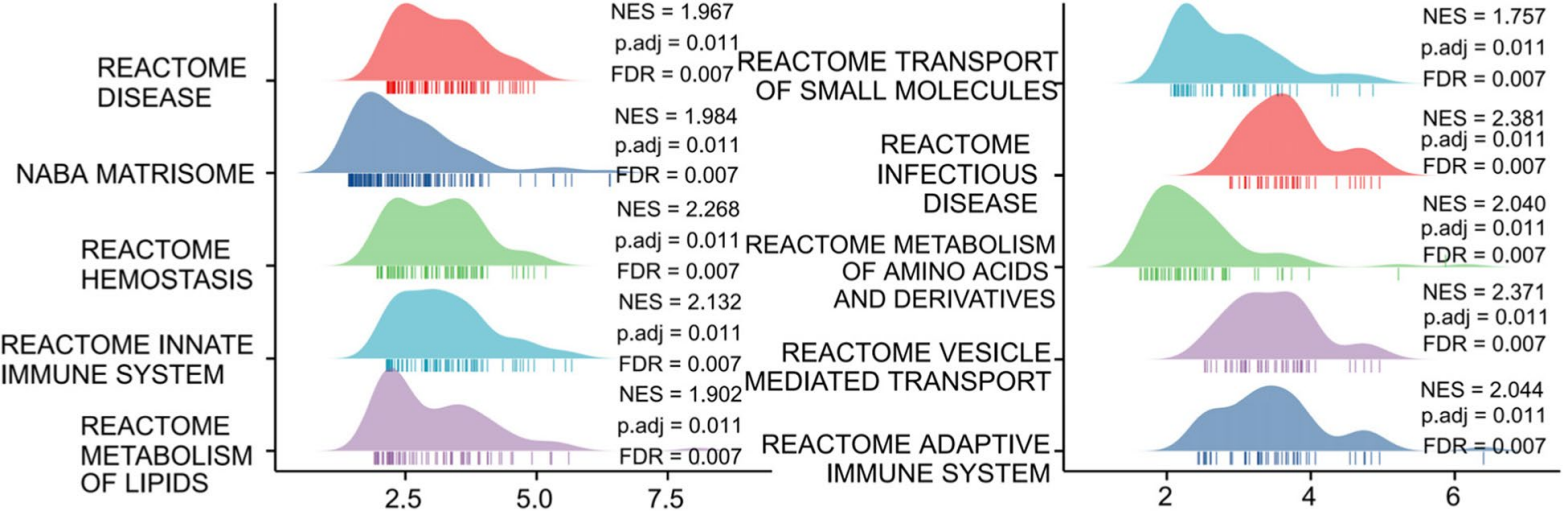
GSEA analysis of the DEGs between the high-risk group and the low-risk group in the TCGA-HCC cohort. Top 10 terms of GSEA analysis (Reactome disease, NABA matrisome, Hemostasis, Innate immune system, Metabolism of lipids, Transport of small molecules, Infectious disease, Metabolism of amion acids and derivatives, Vesicle mediated transport, Adaptive immune system)

To reveal the potential impact of immune infiltration-related genes on the occurrence and development of HCC, we performed GSEA on the DEGs between the high-risk group and the low-risk group. GSEA showed that the DEGs between the high-risk group and low-risk group were mainly enriched in several pathways, including disease, matrisome, haemostasis, innate immune system, metabolism of lipids, transport of small molecules, infectious disease, metabolism of amino acids and derivatives, vesicle-mediated transport, and adaptive immune system (Fig. 7). These findings suggested that immune infiltration-related genes may play a potential role in amino acid and lipid metabolism, matrisome and small molecule transportation, immune system regulation, and hepatitis virus infection in HCC.

### Immune cell infiltration level analysis

**Fig. 8:**
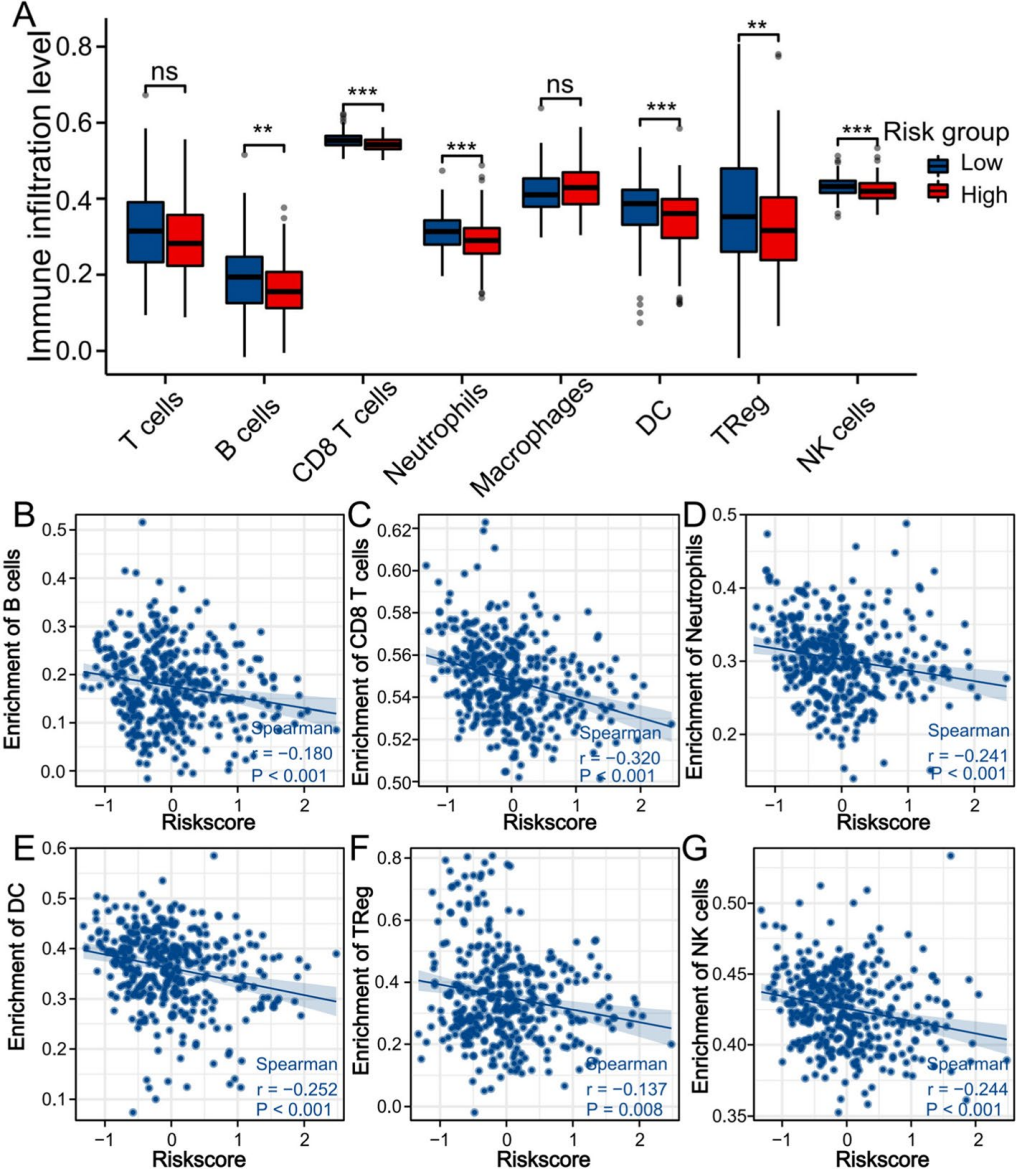
Analysis of immune cell infiltration in TCGA-HCC cohort. **A** The box plot showed the levels of immune cell infiltration between the high-risk group and low-risk group in HCC patients. Scatter plots of correlation between immune cell infiltrations and risk score (**B**, B cells; **C**, CD8 T cells; **D**, Neutrophils; **E**, DC; **F**, TReg; **G**, NK cells). **P < 0.01, ***P < 0.001, ns, not significant

We also calculated the correlation between this prognostic model based on patients in the TCGA-HCC cohort and immune cell infiltration. The results showed that the high-risk group showed lower levels of immune cell infiltration, such as B cells (*P* < 0.01), CD8 T cells (*P* < 0.001), neutrophils (*P* < 0.001), DCs (*P* < 0.001), Tregs (*P* < 0.01), and NK cells (*P* < 0.001) (Fig. 8A). Moreover, the risk score was negatively correlated with infiltrating immune cells, including B cells, CD8 T cells, neutrophils, DCs, Tregs, and NK cells (Fig. 8B-G).

## Discussion

The onset of HCC is insidious, and clinical symptoms often occur when the disease has progressed to the middle and late stages [17]. Because of its high malignancy and insensitivity to radiotherapy and chemotherapy, the prognosis of HCC patients is poor [2]. As an important part of the tumour immune microenvironment, tumour immune infiltration has been proven to have good prognostic value in many solid tumours [18–20]. Immune infiltration-related genes are the molecular basis of tumour immune infiltration, and their importance in elucidating the mechanism of tumorigenesis and development has been confirmed in a number of studies in recent years [8, 9]. However, the prognostic value of immune infiltration-related genes in HCC still needs to be further studied.

In our research, we downloaded gene expression data and clinical information from the TCGA database. After DEG screening and immune infiltration-related gene comparison, we selected 89 immune infiltration-related DEGs. Among them, 17 genes were identified as potential prognostic markers through univariate Cox regression analysis and LASSO regression analysis. Subsequently, we used these 17 immune infiltration-related genes to construct a prognostic model. Among them, the expression levels of 12 genes (ORC1, VNN2, SKA1, BRSK2, MSC, CCR3, DACH1, TNFRSF4, RPL10L, CDC25A, BACH2, and MMP9) were negatively correlated with OS, and the expression levels of 5 genes (STEAP4, IGHM, CYP27A1, REN, and CD4) were positively correlated with OS (Fig. S3). By multivariate Cox regression analysis, we also verified the effectiveness and stability of the model in TCGA-HCC patients and its reliability as an independent predictor of OS in HCC patients. Moreover, HCC patients with high histological grade, high T stage, residual tumour, high pathological stage, vascular invasion, and AFP > 400 had higher risk scores; these characteristics often indicate that the disease is more serious in HCC patients. Studies have shown that the tumour immune microenvironment of HCC patients with rapid progression is often associated with poor immune cell infiltration. This result strongly confirms the above conclusion.

Studies have shown that immune infiltration-related genes are involved in the pathological process of HCC. ORC1 is an important origin recognition complex subunit. Wang XK et al. found that ORC1 was highly expressed in HCC and played an important role in the survival prediction and recurrence monitoring of HCC [21]. VNN2 is mainly involved in hydrolase activity, and its product is a member of the Vanin protein family. Li W et al. determined the prognostic value of a predictive six-gene model including VNN2 in HCC by means of bioinformatics analysis [22]. MSC can encode transcriptional inhibitors and is the downstream target of the B-cell receptor signal transduction pathway. Zhang FP et al. demonstrated that HCC patients might benefit from individualized immunotherapy by establishing an eight-gene risk score model including MSC [23]. CCR3 can encode C-C chemokine receptors and is highly expressed in eosinophils and basophils. A study showed that tumour necrosis factor-α (TNF-α) can significantly induce IL-8 production in HCC cells by inhibiting CCR3. Therefore, CCR3 might be a potential target for the treatment and prognostic guidance of HCC patients [24]. DACH1 encodes a chromatin-related protein, and its expression is lost in some forms of metastatic cancer and is associated with poor prognosis. Research by Qi Cheng et al. showed that DACH1 can affect the proliferation and apoptosis of HCC by regulating p53 [25]. The protein encoded by TNFRSF4 is a member of the TNF receptor superfamily. The receptor has a wide range of biological functions. Research by Xiaoyun Chen et al. showed that TNFRSF4 plays an important role in predicting the early response of HCC to immunotherapy [26]. CDC25A is a member of the CDC25 phosphatase family. CDC25A is necessary for the cell cycle to enter the S phase from the G1 phase. A study showed that inhibiting the activity of CDC25A may provide a new treatment for the control of liver cancer [27]. MMP9 is an important member of the matrix metalloproteinase family, and mouse studies have shown that this enzyme plays a role in tumour-related tissue remodelling. Yujie Ji et al. used nanofibres to deliver chemotherapeutic drugs to inhibit MMP9 to achieve the goal of controlling the progression of HCC [28].

STEAP4 functions as a metalloreductase and may be involved in adipocyte development and metabolism. A study showed that STEAP4 is significantly hypermethylated in HCC tumours, and its epigenetic silencing may be related to HCC [29]. IGHM is an antigen recognition molecule of B cells. The study of Sajjad Karim et al. showed that radiotherapy in cancer patients can cause the downregulation of IGHM expression [30]. CYP27A1 encodes a member of the cytochrome P450 enzyme superfamily. It can catalyse many reactions involving drug metabolism and the synthesis of cholesterol, steroids and other lipids. A study showed that CYP27A1 can be used as a biomarker for HCC progression and a molecular target for the treatment of HCV-related HCC [31]. CD4 encodes the membrane glycoprotein of T lymphocytes and is expressed in T lymphocytes, B cells, macrophages and granulocytes. Its main function is to initiate or enhance the early stages of T-cell activation. A study showed that a high CD4 percentage and high CD4/CD8 ratio affect the OS of HCC patients [32].

The conclusions of the above studies are consistent with the conclusions of this study, highlighting the role of immune infiltration-related genes in the prognosis of HCC. In this study, immune infiltration-related genes were comprehensively analysed, and a risk score model was constructed and validated in the TCGA-HCC cohort. The results showed that the risk scoring model could accurately predict the 1-, 3-, and 5-year OS of HCC patients in the TCGA-HCC cohort.

In addition, to predict the OS of HCC patients, we constructed a prognostic nomogram model based on the immune infiltration-related genes. We incorporated T stage, tumour status and risk group into the nomogram model. The ROC analysis and calibration plots showed that the OS nomogram of the TCGA-HCC cohort has reliable predictive value. The nomogram model we built can be used for the prognostic evaluation and follow-up guidance of HCC patients.

The GSEA results indicated that these immune infiltration-related genes may play a potential role in amino acid and lipid metabolism, matrisome and small molecule transportation, immune system regulation, and hepatitis virus infection in HCC. Avlant Nilsson et al. linked glutamate excretion and nucleotide synthesis to quantitatively analyse amino acid metabolism in HCC and pointed out potential drug targets for HCC [33]. A study showed that dual modification with liposomes provides a potential advantage strategy for the treatment of liver cancer or other liver diseases [34]. Manny D Bacolod et al. found that the activation of T cells and other immune signalling pathways is related to the good prognosis of HCC [35]. Hepatitis B virus (HBV) infection is most likely to contribute to HCC. Yuqin Tang et al. identified new tumour biomarkers with potential targeted therapy effects related to HBV-related HCC by bioinformatics analysis [36]. The above studies have well confirmed the reliability of our findings, but the specific mechanism of each pathway in HCC still needs to be further studied.

The immune cell infiltration analysis showed that the level of immune cell infiltration in the high-risk group in the TCGA-HCC cohort was low, and the risk score was negatively correlated with infiltrating immune cells. Shaoqing Liu et al. found that the infiltration levels of B cells and CD8+ T cells are related to the improvement of OS in HCC patients [37]. HCC usually develops in the context of chronic inflammation [38]. Dalong Ni et al. found that reducing the recruitment and infiltration of neutrophils can inhibit the inflammatory response of the liver, which may reduce the occurrence of HCC [39]. A phase I/IIa study conducted by Jeong-Hoon Lee et al. showed that the adjuvant DC vaccine for HCC is safe and well tolerated and can effectively improve the prognosis of LICH patients [40]. A study found that the infiltration levels of Treg cells and NK cells in HCC tumour tissues are low and are significantly related to the prognosis of HCC patients [41]. It is well known that changes in the immune microenvironment are closely related to the occurrence and development of tumours. The above studies all proposed the effect of immune infiltrating cells on HCC through the study of a certain immune cell. Our study systematically evaluated the infiltration of immune cells in HCC through immune infiltration-related genes, which provides new ideas and methods for the study of immune infiltration in HCC.

## Conclusion

In summary, we created and validated a risk scoring system based on immune infiltration-related genes that was derived from the TCGA data set for the prognostic assessment and risk stratification of HCC patients. A nomogram model for 1-year, 3-year and 5-year OS predictions was established, and it had good predictive accuracy. The 17 genes in the risk score we established might become potential targets for understanding the biological mechanisms of HCC. In addition, GSEA and tumour immune infiltration analysis indicated that immune infiltration-related genes may be involved in biological processes such as amino acid and lipid metabolism, matrisome and small molecule transportation, immune system regulation, and hepatitis virus infection. These results might provide new ideas for HCC research. However, the above conclusions were all drawn from bioinformatics analysis and still need to be verified by a large sample of prospective studies.

## Supplementary Information

**Additional file 1: Table S1**. Immune infiltration-related genes.

**Additional file 2: Table S1**. The complete results of GO and KEGG analysis.

**Additional file 3: Table S3.** 27 immune infiltration-related genes related to OS.

**Additional file 4: Figure S1**. Heat map of 89 DEGs related to immune infiltration in the data set GSE87630.

**Additional file 5: Figure S2.** Heat map of 89 DEGs related to immune infiltration in the data set GSE89377.

**Additional file 6: Figure S3**. KM survival curves of the 17 immune infiltration-related genes. (A) ORC1. (B) VNN2. (C) STEAP4. (D) SKA1. (E) BRSK2. (F) MSC. (G) CCR3. (H) IGHM. (I) CYP27A1. (J) DACH1. (K) TNFRSF4. (L) RPL10L. (M) CDC25A. (N) REN. (O) BACH2. (P) MMP9. (Q) CD4.

## Data Availability

The data sets analysed during the current study are all available in public databases. The gene expression data and clinical data of 374 HCC samples and 50 control samples were obtained from the TCGA database (https://portal.gdc.cancer.gov/). The immune infiltration-related gene expression validation data sets GSE25097, GSE87630 and GSE89377 were obtained from the GEO database (https://www.ncbi.nlm.nih.gov/geo/). The immune infiltration-related gene data were downloaded from CIBERSORTX (https://cibersortx.stanford.edu/). The immunohistochemical data of immune infiltration-related genes in HCC and normal liver tissues were obtained from the Human Protein Atlas (HPA) database (https://www.proteinatlas.org/).

## Abbreviations

HCC: Hepatocellular carcinoma
TCGA: The Cancer Genome Atlas
LASSO: Least absolute shrinkage and selection operator
KM: Kaplan–Meier
ROC: Receiver operating characteristic
GSEA: Gene set enrichment analysis
OS: Overall survival
GEO: Gene Expression Omnibus
DEGs: Differentially expressed genes
HR: Hazard ratio
CI: Confidence interval
KEGG: Kyoto Encyclopedia of Genes and Genomes
HPA: Human Protein Atlas

## References

[1] Yang W, Ma Y, Liu Y (2019). Association of intake of whole grains and dietary fiber with risk of hepatocellular carcinoma in US adults. JAMA Oncol..

[2] Shen X, Hu B, Xu J (2020). The m6A methylation landscape stratifies hepatocellular carcinoma into 3 subtypes with distinct metabolic characteristics. Cancer Biol Med..

[3] Xu Q, Li Y, Gao X (2020). HNF4α regulates sulfur amino acid metabolism and confers sensitivity to methionine restriction in liver cancer. Nat Commun..

[4] Huang X, Gan G, Wang X (2019). The HGF-MET axis coordinates liver cancer metabolism and autophagy for chemotherapeutic resistance. Autophagy..

[5] McCulloch K, Romero N, MacLachlan J (2020). Modeling progress toward elimination of Hepatitis B in Australia. Hepatology..

[6] Zuo S, Wei M, Wang S (2020). Pan-cancer analysis of immune cell infiltration identifies a prognostic Immune-Cell Characteristic Score (ICCS) in Lung Adenocarcinoma. Front Immunol..

[7] Mo X, Huang X, Feng Y (2020). Immune infiltration and immune gene signature predict the response to fluoropyrimidine-based chemotherapy in colorectal cancer patients. Oncoimmunology..

[8] Shahamatdar S, He MX, Reyna MA (2020). Germline features associated with immune infiltration in solid tumors. Cell Rep..

[9] Li Y, Burgman B, McGrail DJ (2020). Integrated genomic characterization of the human immunome in cancer. Cancer Res..

[10] Zhang X, Shi M, Chen T (2020). Characterization of the immune cell infiltration landscape in head and neck squamous cell carcinoma to aid immunotherapy. Mol Ther Nucleic Acids..

[11] Love MI, Huber W, Anders S (2014). Moderated estimation of fold change and dispersion for RNA-seq data with DESeq2. Genome biology.

[12] Yu G, Wang LG, Han Y (2012). clusterProfiler: an R package for comparing biological themes among gene clusters. OMICS..

[13] Walter W, Sánchez-Cabo F, Ricote M (2015). GOplot: an R package for visually combining expression data with functional analysis. Bioinformatics.

[14] Xu S, Wang Z, Ye J, Mei S, Zhang J (2021). Identification of iron metabolism-related genes as prognostic indicators for lower-grade glioma. Front Oncol..

[15] Hänzelmann S, Castelo R, Guinney J (2013). GSVA: gene set variation analysis for microarray and RNA-seq data. BMC Bioinformatics.

[16] Bindea G (2013). Spatiotemporal dynamics of intratumoral immune cells reveal the immune landscape in human cancer. Immunity.

[17] Mikami D, Kobayashi M, Uwada J (2020). AR420626, a selective agonist of GPR41/FFA3, suppresses growth of hepatocellular carcinoma cells by inducing apoptosis via HDAC inhibition. Ther Adv Med Oncol..

[18] Finotello F, Mayer C, Plattner C (2019). Molecular and pharmacological modulators of the tumor immune contexture revealed by deconvolution of RNA-seq data [published correction appears in Genome Med. 2019 Jul 29;11(1):50]. Genome Med.

[19] Loi S, Drubay D, Adams S (2019). Tumor-infiltrating lymphocytes and prognosis: a pooled individual patient analysis of early-stage triple-negative breast cancers. J Clin Oncol..

[20] Li R, Liu H, Cao Y (2020). Identification and validation of an immunogenic subtype of gastric cancer with abundant intratumoural CD103+CD8+ T cells conferring favourable prognosis. Br J Cancer..

[21] Wang XK, Wang QQ, Huang JL (2020). Novel candidate biomarkers of origin recognition complex 1, 5 and 6 for survival surveillance in patients with hepatocellular carcinoma. J Cancer..

[22] Li W, Lu J, Ma Z (2020). An integrated model based on a six-gene signature predicts overall survival in patients with hepatocellular carcinoma. Front Genet..

[23] Zhang FP, Huang YP, Luo WX (2020). Construction of a risk score prognosis model based on hepatocellular carcinoma microenvironment. World J Gastroenterol..

[24] Wang Y, Wang W, Wang L (2012). Regulatory mechanisms of interleukin-8 production induced by tumour necrosis factor-α in human hepatocellular carcinoma cells. J Cell Mol Med..

[25] Cheng Q, Ning D, Chen J (2018). SIX1 and DACH1 influence the proliferation and apoptosis of hepatocellular carcinoma through regulating p53. Cancer Biol Ther..

[26] Chen X, Li D, Cao Y (2019). Early therapeutic vaccination prediction of hepatocellular carcinoma via imaging OX40-mediated tumor infiltrating lymphocytes. Mol Pharm..

[27] Wang Z, Kar S, Carr BI (2008). Cdc25A protein phosphatase: a therapeutic target for liver cancer therapies. Anticancer Agents Med Chem..

[28] Ji Y, Xiao Y, Xu L (2018). Drug-bearing supramolecular MMP inhibitor nanofibers for inhibition of metastasis and growth of liver cancer. Adv Sci (Weinh)..

[29] Yamada N, Yasui K, Dohi O (2016). Genome-wide DNA methylation analysis in hepatocellular carcinoma. Oncol Rep..

[30] Karim S, Mirza Z, Chaudhary AG (2016). Assessment of radiation induced therapeutic effect and cytotoxicity in cancer patients based on transcriptomic profiling. Int J Mol Sci..

[31] Tsunedomi R, Iizuka N, Hamamoto Y (2005). Patterns of expression of cytochrome P450 genes in progression of hepatitis C virus-associated hepatocellular carcinoma. Int J Oncol..

[32] El-Rebey HS, Abdou AG, Sultan MM (2021). The profile and role of tumor-infiltrating lymphocytes in hepatocellular carcinoma: an immunohistochemical study. Appl Immunohistochem Mol Morphol..

[33] Nilsson A, Haanstra JR, Engqvist M (2020). Quantitative analysis of amino acid metabolism in liver cancer links glutamate excretion to nucleotide synthesis. Proc Natl Acad Sci U S A..

[34] Wang S, Xu H, Xu J (2010). Sustained liver targeting and improved antiproliferative effect of doxorubicin liposomes modified with galactosylated lipid and PEG-lipid. AAPS PharmSciTech..

[35] Bacolod MD, Barany F, Pilones K (2019). Pathways- and epigenetic-based assessment of relative immune infiltration in various types of solid tumors. Adv Cancer Res..

[36] Tang Y, Zhang Y, Hu X (2020). Identification of potential hub genes related to diagnosis and prognosis of Hepatitis B virus-related hepatocellular carcinoma via integrated bioinformatics analysis. Biomed Res Int..

[37] Liu S, Tang Q, Huang J (2021). Prognostic analysis of tumor mutation burden and immune infiltration in hepatocellular carcinoma based on TCGA data. Aging (Albany NY)..

[38] Berasain C, Perugorria MJ, Latasa MU (2009). The epidermal growth factor receptor: a link between inflammation and liver cancer. Exp Biol Med (Maywood)..

[39] Ni D, Wei H, Chen W (2019). Ceria nanoparticles meet hepatic ischemia-reperfusion injury: the perfect imperfection. Adv Mater..

[40] Lee JH, Lee Y, Lee M (2015). A phase I/IIa study of adjuvant immunotherapy with tumour antigen-pulsed dendritic cells in patients with hepatocellular carcinoma. Br J Cancer..

[41] Guo CL, Yang HC, Yang XH (2012). Associations between infiltrating lymphocyte subsets and hepatocellular carcinoma. Asian Pac J Cancer Prev..

