# Supplementary material for: Identification of immune infiltration-related genes as prognostic indicators for hepatocellular carcinoma
Source: BMC Cancer. 2022 May 5;22:496. doi: 10.1186/s12885-022-09587-0 (PMC9074323; doi:10.1186/s12885-022-09587-0)
Supplement: Supplementary file 1 — Additional file 1: Table S1. Immune infiltration-related genes. [file 12885_2022_9587_MOESM1_ESM.docx]

**Table S1. Immune infiltration-related genes.**

| **Immune infiltration-related gene list** | | | | | |
| --- | --- | --- | --- | --- | --- |
| ABCB4 | CD33 | EMR2 | IL12B | NAALADL1 | SKA1 |
| ABCB9 | CD37 | EMR3 | IL12RB2 | NCF2 | SKAP1 |
| ACAP1 | CD38 | EPB41 | IL17A | NCR3 | SLAMF1 |
| ACHE | CD3D | EPHA1 | IL18R1 | NFE2 | SLAMF8 |
| ACP5 | CD3E | EPN2 | IL18RAP | NIPSNAP3B | SLC12A1 |
| ADAM28 | CD3G | ETS1 | IL1A | NKG7 | SLC12A8 |
| ADAMDEC1 | CD4 | ETV3 | IL1B | NLRP3 | SLC15A3 |
| ADAMTS3 | CD40 | FAIM3 | IL1RL1 | NMBR | SLC2A6 |
| ADRB2 | CD40LG | FAM124B | IL21 | NME8 | SLC7A10 |
| AIF1 | CD5 | FAM174B | IL26 | NOD2 | SLCO5A1 |
| AIM2 | CD6 | FAM198B | IL2RA | NOX3 | SMPD3 |
| ALOX15 | CD68 | FAM212B | IL2RB | NPAS1 | SMPDL3B |
| ALOX5 | CD69 | FAM65B | IL3 | NPIPB15 | SOCS1 |
| AMPD1 | CD7 | FASLG | IL4 | NPL | SP140 |
| ANGPT4 | CD70 | FBXL8 | IL4R | NR4A3 | SPAG4 |
| ANKRD55 | CD72 | FCER1A | IL5 | NTN3 | SPIB |
| APOBEC3A | CD79A | FCER2 | IL5RA | NTRK1 | SPOCK2 |
| APOBEC3G | CD79B | FCGR2B | IL7 | ORC1 | SSX1 |
| APOL3 | CD80 | FCGR3B | IL7R | OSM | ST3GAL6 |
| APOL6 | CD86 | FCN1 | IL9 | P2RX1 | ST6GALNAC4 |
| AQP9 | CD8A | FCRL2 | IRF8 | P2RX5 | ST8SIA1 |
| ARHGAP22 | CD8B | FES | ITK | P2RY10 | STAP1 |
| ARRB1 | CD96 | FFAR2 | KCNA3 | P2RY13 | STEAP4 |
| ASGR1 | CDA | FLJ13197 | KCNG2 | P2RY14 | STXBP6 |
| ASGR2 | CDC25A | FLT3LG | KIAA0226L | P2RY2 | TARDBPP1 |
| ATHL1 | CDH12 | FLVCR2 | KIAA0754 | PADI4 | TBX21 |
| ATP8B4 | CDHR1 | FOSB | KIR2DL1 | PAQR5 | TCF7 |
| ATXN8OS | CDK6 | FOXP3 | KIR2DL4 | PASK | TCL1A |
| AZU1 | CEACAM3 | FPR1 | KIR2DS4 | PAX7 | TEC |
| BACH2 | CEACAM8 | FPR2 | KIR3DL2 | PBXIP1 | TEP1 |
| BANK1 | CEMP1 | FPR3 | KIRREL | PCDHA5 | TGM5 |
| BARX2 | CFP | FRK | KLRB1 | PDCD1 | TLR2 |
| BCL11B | CHI3L1 | FRMD4A | KLRC3 | PDCD1LG2 | TLR7 |
| BCL2A1 | CHI3L2 | FRMD8 | KLRC4 | PDE6C | TLR8 |
| BCL7A | CHST15 | FZD2 | KLRD1 | PDK1 | TMEM156 |
| BEND5 | CHST7 | FZD3 | KLRF1 | PGLYRP1 | TMEM255A |
| BFSP1 | CLC | GAL3ST4 | KLRG1 | PIK3IP1 | TNFAIP6 |
| BHLHE41 | CLCA3P | GALR1 | KLRK1 | PKD2L2 | TNFRSF10C |
| BIRC3 | CLEC10A | GFI1 | KRT18P50 | PLA1A | TNFRSF11A |
| BLK | CLEC2D | GGT5 | KYNU | PLA2G7 | TNFRSF13B |
| BMP2K | CLEC4A | GIPR | LAG3 | PLCH2 | TNFRSF17 |
| BPI | CLEC7A | GNG7 | LAIR2 | PLEKHF1 | TNFRSF4 |
| BRAF | CLIC2 | GNLY | LAMP3 | PLEKHG3 | TNFSF14 |
| BRSK2 | CMA1 | GPC4 | LAT | PMCH | TNIP3 |
| BST1 | COL8A2 | GPR1 | LCK | PNOC | TPSAB1 |
| BTNL8 | COLQ | GPR171 | LEF1 | PPBP | TRAC |
| C11orf80 | CPA3 | GPR18 | LHCGR | PPFIBP1 | TRAF4 |
| C1orf54 | CR2 | GPR183 | LILRA2 | PRF1 | TRAT1 |
| C3AR1 | CREB5 | GPR19 | LILRA3 | PRG2 | TRAV12-2 |
| C5AR1 | CRISP3 | GPR25 | LILRA4 | PRR5L | TRAV13-1 |
| C5AR2 | CRTAM | GPR65 | LILRB2 | PSG2 | TRAV13-2 |
| CA8 | CRYBB1 | GPR97 | LIME1 | PTGDR | TRAV21 |
| CAMP | CSF1 | GRAP2 | LINC00597 | PTGER2 | TRAV8-6 |
| CASP5 | CSF2 | GSTT1 | LINC00921 | PTGIR | TRAV9-2 |
| CCDC102B | CSF3R | GUSBP11 | LOC100130100 | PTPRCAP | TRBC1 |
| CCL1 | CST7 | GYPE | LOC126987 | PTPRG | TRDC |
| CCL13 | CTLA4 | GZMA | LRMP | PVRIG | TREM1 |
| CCL14 | CTSG | GZMB | LST1 | QPCT | TREM2 |
| CCL17 | CTSW | GZMH | LTA | RAB27B | TREML2 |
| CCL18 | CXCL10 | GZMK | LTB | RALGPS2 | TRIB2 |
| CCL19 | CXCL11 | GZMM | LTC4S | RASA3 | TRPM4 |
| CCL20 | CXCL13 | HAL | LY86 | RASGRP2 | TRPM6 |
| CCL22 | CXCL3 | HCK | LY9 | RASGRP3 | TSHR |
| CCL23 | CXCL5 | HDC | MAGEA11 | RASSF4 | TTC38 |
| CCL4 | CXCL9 | HESX1 | MAK | RCAN3 | TXK |
| CCL5 | CXCR1 | HHEX | MAN1A1 | REN | TYR |
| CCL7 | CXCR2 | HIC1 | MANEA | RENBP | UBASH3A |
| CCL8 | CXCR5 | HIST1H2AE | MAP3K13 | REPS2 | UGT1A8 |
| CCND2 | CXCR6 | HIST1H2BG | MAP4K1 | RGS1 | UGT2B17 |
| CCR10 | CXorf57 | HK3 | MAP4K2 | RGS13 | UPK3A |
| CCR2 | CYP27A1 | HLA-DOB | MAP9 | RNASE2 | VILL |
| CCR3 | CYP27B1 | HLA-DQA1 | 3-Mar | RNASE6 | VNN1 |
| CCR5 | DACH1 | HMGB3P30 | MARCO | RPL10L | VNN2 |
| CCR6 | DAPK2 | HNMT | MAST1 | RPL3P7 | VNN3 |
| CCR7 | DCSTAMP | HOXA1 | MBL2 | RRP12 | VPREB3 |
| CD160 | DEFA4 | HPGDS | MEFV | RRP9 | WNT5B |
| CD180 | DENND5B | HPSE | MEP1A | RSAD2 | WNT7A |
| CD19 | DEPDC5 | HRH1 | MGAM | RYR1 | ZAP70 |
| CD1A | DGKA | HSPA6 | MICAL3 | S100A12 | ZBP1 |
| CD1B | DHRS11 | HTR2B | MMP12 | S1PR5 | ZBTB10 |
| CD1C | DHX58 | ICA1 | MMP25 | SAMSN1 | ZBTB32 |
| CD1D | DPEP2 | ICOS | MMP9 | SCN9A | ZFP36L2 |
| CD1E | DPP4 | IDO1 | MNDA | SEC31B | ZNF135 |
| CD2 | DSC1 | IFI44L | MROH7 | SELL | ZNF165 |
| CD209 | DUSP2 | IFNA10 | MS4A1 | 5-Sep | ZNF204P |
| CD22 | EAF2 | IFNG | MS4A2 | 8-Sep | ZNF222 |
| CD244 | EBI3 | IGHD | MS4A3 | SERGEF | ZNF286A |
| CD247 | EFNA5 | IGHE | MS4A6A | SH2D1A | ZNF324 |
| CD27 | EGR2 | IGHM | MSC | SIGLEC1 | ZNF442 |
| CD28 | ELANE | IGKC | MXD1 | SIK1 | MZB1 |
| CD300A | EMR1 | IGLL3P | MYB | SIRPG | SIT1 |
| ABCB4 | CD33 | IGSF6 |  |  |  |
