# Supplementary material for: Identification of immune infiltration-related genes as prognostic indicators for hepatocellular carcinoma
Source: BMC Cancer. 2022 May 5;22:496. doi: 10.1186/s12885-022-09587-0 (PMC9074323; doi:10.1186/s12885-022-09587-0)
Supplement: Supplementary file 2 — Additional file 2: Table S1. The complete results of GO and KEGG analysis. [file 12885_2022_9587_MOESM2_ESM.docx]

**Table S2. The complete results of GO and KEGG analysis**

| ONTOLOGY | ID | Description | GeneRatio | BgRatio | p.adjust | qvalue | zscore |
| --- | --- | --- | --- | --- | --- | --- | --- |
| BP | GO:0030593 | neutrophil chemotaxis | 11/82 | 104/18670 | 1.96663E-09 | 1.59219E-09 | -0.904534034 |
| BP | GO:1990266 | neutrophil migration | 11/82 | 118/18670 | 2.49914E-09 | 2.0233E-09 | -0.904534034 |
| BP | GO:0050900 | leukocyte migration | 18/82 | 499/18670 | 2.49914E-09 | 2.0233E-09 | -1.414213562 |
| BP | GO:0070098 | chemokine-mediated signaling pathway | 10/82 | 88/18670 | 2.49914E-09 | 2.0233E-09 | 0 |
| BP | GO:0071621 | granulocyte chemotaxis | 11/82 | 123/18670 | 2.49914E-09 | 2.0233E-09 | -0.904534034 |
| BP | GO:0097529 | myeloid leukocyte migration | 13/82 | 210/18670 | 2.49914E-09 | 2.0233E-09 | -0.832050294 |
| BP | GO:1990868 | response to chemokine | 10/82 | 97/18670 | 3.66257E-09 | 2.96522E-09 | 0 |
| BP | GO:1990869 | cellular response to chemokine | 10/82 | 97/18670 | 3.66257E-09 | 2.96522E-09 | 0 |
| BP | GO:0097530 | granulocyte migration | 11/82 | 141/18670 | 6.32806E-09 | 5.1232E-09 | -0.904534034 |
| BP | GO:0060326 | cell chemotaxis | 14/82 | 304/18670 | 1.16345E-08 | 9.41927E-09 | -0.534522484 |
| BP | GO:0030595 | leukocyte chemotaxis | 12/82 | 224/18670 | 4.98494E-08 | 4.03581E-08 | -1.154700538 |
| BP | GO:0042742 | defense response to bacterium | 13/82 | 330/18670 | 3.37677E-07 | 2.73383E-07 | -2.496150883 |
| BP | GO:0042119 | neutrophil activation | 14/82 | 498/18670 | 5.10514E-06 | 4.13312E-06 | -2.672612419 |
| BP | GO:0002819 | regulation of adaptive immune response | 9/82 | 160/18670 | 5.10514E-06 | 4.13312E-06 | -3 |
| BP | GO:0006959 | humoral immune response | 12/82 | 356/18670 | 6.69573E-06 | 5.42086E-06 | -1.732050808 |
| BP | GO:0050663 | cytokine secretion | 10/82 | 240/18670 | 1.27105E-05 | 1.02904E-05 | -1.897366596 |
| BP | GO:0050708 | regulation of protein secretion | 13/82 | 472/18670 | 1.63937E-05 | 1.32724E-05 | -0.832050294 |
| BP | GO:0002700 | regulation of production of molecular mediator of immune response | 8/82 | 139/18670 | 2.00836E-05 | 1.62597E-05 | -2.121320344 |
| BP | GO:0002283 | neutrophil activation involved in immune response | 13/82 | 488/18670 | 2.15147E-05 | 1.74183E-05 | -2.496150883 |
| BP | GO:0002791 | regulation of peptide secretion | 13/82 | 500/18670 | 2.69844E-05 | 2.18466E-05 | -0.832050294 |
| BP | GO:0050714 | positive regulation of protein secretion | 10/82 | 268/18670 | 2.69844E-05 | 2.18466E-05 | -0.632455532 |
| BP | GO:0050707 | regulation of cytokine secretion | 9/82 | 210/18670 | 3.32578E-05 | 2.69255E-05 | -1.666666667 |
| BP | GO:0002548 | monocyte chemotaxis | 6/82 | 65/18670 | 3.64228E-05 | 2.94879E-05 | -0.816496581 |
| BP | GO:0002460 | adaptive immune response based on somatic recombination of immune receptors built from immunoglobulin superfamily domains | 11/82 | 361/18670 | 4.34235E-05 | 3.51557E-05 | -2.713602101 |
| BP | GO:0002793 | positive regulation of peptide secretion | 10/82 | 288/18670 | 4.39025E-05 | 3.55435E-05 | -0.632455532 |
| BP | GO:0002697 | regulation of immune effector process | 12/82 | 458/18670 | 5.7926E-05 | 4.6897E-05 | -2.309401077 |
| BP | GO:0001819 | positive regulation of cytokine production | 12/82 | 464/18670 | 6.39718E-05 | 5.17916E-05 | -2.309401077 |
| BP | GO:0055074 | calcium ion homeostasis | 12/82 | 471/18670 | 7.2192E-05 | 5.84467E-05 | -0.577350269 |
| BP | GO:0050867 | positive regulation of cell activation | 11/82 | 394/18670 | 8.48357E-05 | 6.8683E-05 | -2.110579412 |
| BP | GO:0043312 | neutrophil degranulation | 12/82 | 485/18670 | 8.97386E-05 | 7.26524E-05 | -2.309401077 |
| BP | GO:1903532 | positive regulation of secretion by cell | 11/82 | 399/18670 | 8.97386E-05 | 7.26524E-05 | -0.904534034 |
| BP | GO:0002446 | neutrophil mediated immunity | 12/82 | 499/18670 | 0.000115401 | 9.34288E-05 | -2.309401077 |
| BP | GO:0050715 | positive regulation of cytokine secretion | 7/82 | 139/18670 | 0.000161351 | 0.00013063 | -1.889822365 |
| BP | GO:0051047 | positive regulation of secretion | 11/82 | 428/18670 | 0.000161351 | 0.00013063 | -0.904534034 |
| BP | GO:0071674 | mononuclear cell migration | 6/82 | 90/18670 | 0.000165346 | 0.000133864 | -0.816496581 |
| BP | GO:0002822 | regulation of adaptive immune response based on somatic recombination of immune receptors built from immunoglobulin superfamily domains | 7/82 | 145/18670 | 0.000200206 | 0.000162087 | -2.645751311 |
| BP | GO:0002702 | positive regulation of production of molecular mediator of immune response | 6/82 | 95/18670 | 0.000212443 | 0.000171994 | -1.632993162 |
| BP | GO:1904645 | response to amyloid-beta | 5/82 | 54/18670 | 0.000212443 | 0.000171994 | 0.447213595 |
| BP | GO:0070374 | positive regulation of ERK1 and ERK2 cascade | 8/82 | 215/18670 | 0.000249889 | 0.00020231 | -0.707106781 |
| BP | GO:0006874 | cellular calcium ion homeostasis | 11/82 | 458/18670 | 0.000262175 | 0.000212257 | -0.904534034 |
| BP | GO:0032649 | regulation of interferon-gamma production | 6/82 | 101/18670 | 0.000276498 | 0.000223853 | -2.449489743 |
| BP | GO:0002696 | positive regulation of leukocyte activation | 10/82 | 380/18670 | 0.000313827 | 0.000254074 | -1.897366596 |
| BP | GO:0070372 | regulation of ERK1 and ERK2 cascade | 9/82 | 300/18670 | 0.000323323 | 0.000261762 | -1 |
| BP | GO:0002429 | immune response-activating cell surface receptor signaling pathway | 11/82 | 473/18670 | 0.000323478 | 0.000261888 | -2.713602101 |
| BP | GO:0001906 | cell killing | 7/82 | 168/18670 | 0.000422562 | 0.000342106 | -1.889822365 |
| BP | GO:0072503 | cellular divalent inorganic cation homeostasis | 11/82 | 493/18670 | 0.000451547 | 0.000365573 | -0.904534034 |
| BP | GO:0070371 | ERK1 and ERK2 cascade | 9/82 | 317/18670 | 0.000451547 | 0.000365573 | -1 |
| BP | GO:0032609 | interferon-gamma production | 6/82 | 113/18670 | 0.000451547 | 0.000365573 | -2.449489743 |
| CC | GO:0009897 | external side of plasma membrane | 12/84 | 393/19717 | 1.22815E-05 | 1.11897E-05 | -1.732050808 |
| CC | GO:1904724 | tertiary granule lumen | 5/84 | 55/19717 | 0.000217849 | 0.000198483 | -0.447213595 |
| CC | GO:0070820 | tertiary granule | 7/84 | 164/19717 | 0.000254286 | 0.000231681 | -1.133893419 |
| CC | GO:0042581 | specific granule | 6/84 | 160/19717 | 0.001864745 | 0.001698971 | -1.632993162 |
| CC | GO:0030667 | secretory granule membrane | 6/84 | 298/19717 | 0.040867184 | 0.037234143 | -2.449489743 |
| CC | GO:0035580 | specific granule lumen | 3/84 | 62/19717 | 0.046680779 | 0.042530917 | -0.577350269 |
| MF | GO:0008009 | chemokine activity | 6/82 | 49/17697 | 2.30364E-05 | 1.92959E-05 | 0 |
| MF | GO:0042379 | chemokine receptor binding | 6/82 | 66/17697 | 7.02959E-05 | 5.88817E-05 | 0 |
| MF | GO:0001664 | G protein-coupled receptor binding | 9/82 | 280/17697 | 0.000457142 | 0.000382914 | -1 |
| MF | GO:0005125 | cytokine activity | 8/82 | 220/17697 | 0.000477371 | 0.000399858 | -0.707106781 |
| MF | GO:0048020 | CCR chemokine receptor binding | 4/82 | 43/17697 | 0.002088681 | 0.001749533 | 0 |
| MF | GO:0008528 | G protein-coupled peptide receptor activity | 6/82 | 146/17697 | 0.002088681 | 0.001749533 | -0.816496581 |
| MF | GO:0042834 | peptidoglycan binding | 3/82 | 17/17697 | 0.002088681 | 0.001749533 | -0.577350269 |
| MF | GO:0001653 | peptide receptor activity | 6/82 | 152/17697 | 0.002166091 | 0.001814374 | -0.816496581 |
| MF | GO:0004896 | cytokine receptor activity | 5/82 | 96/17697 | 0.002166091 | 0.001814374 | -1.341640786 |
| MF | GO:0016493 | C-C chemokine receptor activity | 3/82 | 23/17697 | 0.003542622 | 0.002967392 | -0.577350269 |
| MF | GO:0019865 | immunoglobulin binding | 3/82 | 24/17697 | 0.003542622 | 0.002967392 | -1.732050808 |
| MF | GO:0019957 | C-C chemokine binding | 3/82 | 24/17697 | 0.003542622 | 0.002967392 | -0.577350269 |
| MF | GO:0001637 | G protein-coupled chemoattractant receptor activity | 3/82 | 26/17697 | 0.003874707 | 0.003245555 | -0.577350269 |
| MF | GO:0004950 | chemokine receptor activity | 3/82 | 26/17697 | 0.003874707 | 0.003245555 | -0.577350269 |
| MF | GO:0016810 | hydrolase activity, acting on carbon-nitrogen (but not peptide) bonds | 5/82 | 123/17697 | 0.004167513 | 0.003490817 | -1.341640786 |
| MF | GO:0019955 | cytokine binding | 5/82 | 128/17697 | 0.004698444 | 0.003935539 | -1.341640786 |
| MF | GO:0005126 | cytokine receptor binding | 7/82 | 286/17697 | 0.004997728 | 0.004186227 | -0.377964473 |
| MF | GO:0019956 | chemokine binding | 3/82 | 32/17697 | 0.005385029 | 0.00451064 | -0.577350269 |
| MF | GO:0042277 | peptide binding | 7/82 | 295/17697 | 0.005385029 | 0.00451064 | -0.377964473 |
| MF | GO:0001540 | amyloid-beta binding | 4/82 | 78/17697 | 0.005530166 | 0.004632211 | -1 |
| MF | GO:0050786 | RAGE receptor binding | 2/82 | 11/17697 | 0.012704356 | 0.010641499 | -1.414213562 |
| MF | GO:0033218 | amide binding | 7/82 | 356/17697 | 0.01365626 | 0.011438838 | -0.377964473 |
| MF | GO:0004222 | metalloendopeptidase activity | 4/82 | 103/17697 | 0.01365626 | 0.011438838 | 2 |
| MF | GO:0008237 | metallopeptidase activity | 5/82 | 181/17697 | 0.014979572 | 0.012547279 | 2.236067977 |
| MF | GO:0048018 | receptor ligand activity | 8/82 | 482/17697 | 0.01640787 | 0.013743658 | -0.707106781 |
| MF | GO:0008329 | signaling pattern recognition receptor activity | 2/82 | 20/17697 | 0.03450188 | 0.028899671 | -1.414213562 |
| MF | GO:0038187 | pattern recognition receptor activity | 2/82 | 21/17697 | 0.036611294 | 0.030666571 | -1.414213562 |
| KEGG | hsa04061 | Viral protein interaction with cytokine and cytokine receptor | 9/52 | 100/8076 | 1.28307E-06 | 1.19659E-06 | -0.333333333 |
| KEGG | hsa04060 | Cytokine-cytokine receptor interaction | 13/52 | 295/8076 | 1.59228E-06 | 1.48495E-06 | -0.832050294 |
| KEGG | hsa04062 | Chemokine signaling pathway | 9/52 | 192/8076 | 0.000116256 | 0.00010842 | -0.333333333 |
| KEGG | hsa04657 | IL-17 signaling pathway | 5/52 | 94/8076 | 0.009229526 | 0.008607407 | 0.447213595 |
| KEGG | hsa04380 | Osteoclast differentiation | 5/52 | 128/8076 | 0.030185963 | 0.028151267 | -1.341640786 |
| KEGG | hsa05219 | Bladder cancer | 3/52 | 41/8076 | 0.042916484 | 0.040023683 | 1.732050808 |
