# Supplementary material for: Identification of immune infiltration-related genes as prognostic indicators for hepatocellular carcinoma
Source: BMC Cancer. 2022 May 5;22:496. doi: 10.1186/s12885-022-09587-0 (PMC9074323; doi:10.1186/s12885-022-09587-0)
Supplement: Supplementary file 3 — Additional file 3: Table S3. 27 immune infiltration-related genes related to OS. [file 12885_2022_9587_MOESM3_ESM.docx]

**Table S3. 27 immune infiltration-related genes related to OS.**

| Gene name | HR | Lower_95% | Upper_95% | P.value |
| --- | --- | --- | --- | --- |
| CYP27A1 | 0.469 | 0.339 | 0.697 | <0.001 |
| ALOX5 | 1.425 | 1.005 | 2.019 | 0.047 |
| DACH1 | 1.461 | 1.032 | 2.064 | 0.032 |
| CCL23 | 0.673 | 0.475 | 0.954 | 0.026 |
| MMP12 | 1.444 | 1.021 | 2.042 | 0.038 |
| TNFRSF4 | 1.788 | 1.264 | 2.537 | 0.001 |
| GPR1 | 1.63 | 1.148 | 2.314 | 0.006 |
| CCR3 | 2.426 | 1.687 | 3.491 | <0.001 |
| GPR19 | 1.481 | 1.045 | 2.099 | 0.027 |
| STEAP4 | 0.459 | 0.341 | 0.707 | <0.001 |
| IL1B | 1.435 | 1.011 | 2.038 | 0.043 |
| IL1RL1 | 0.692 | 0.488 | 0.979 | 0.038 |
| BTNL8 | 1.448 | 1.022 | 2.052 | 0.037 |
| RPL10L | 1.762 | 1.239 | 2.487 | 0.002 |
| CDC25A | 2.096 | 1.485 | 2.977 | <0.001 |
| CXCL5 | 1.465 | 1.035 | 2.074 | 0.031 |
| SKA1 | 2.094 | 1.482 | 2.964 | <0.001 |
| REN | 0.639 | 0.448 | 0.906 | 0.014 |
| VNN2 | 1.862 | 1.228 | 2.837 | 0.003 |
| BACH2 | 1.485 | 1.053 | 2.114 | 0.024 |
| MMP9 | 1.953 | 1.306 | 2.891 | 0.001 |
| TREM2 | 1.627 | 1.148 | 2.308 | 0.006 |
| ORC1 | 1.842 | 1.314 | 2.613 | 0.001 |
| CD4 | 0.694 | 0.491 | 0.981 | 0.037 |
| BRSK2 | 1.628 | 1.127 | 2.341 | 0.009 |
| MSC | 1.683 | 1.164 | 2.417 | 0.006 |
| IGHM | 0.673 | 0.465 | 0.964 | 0.029 |
