# Supplementary figures and images for: Identification of immune infiltration-related genes as prognostic indicators for hepatocellular carcinoma
Source: BMC Cancer. 2022 May 5;22:496. doi: 10.1186/s12885-022-09587-0 (PMC9074323; doi:10.1186/s12885-022-09587-0)

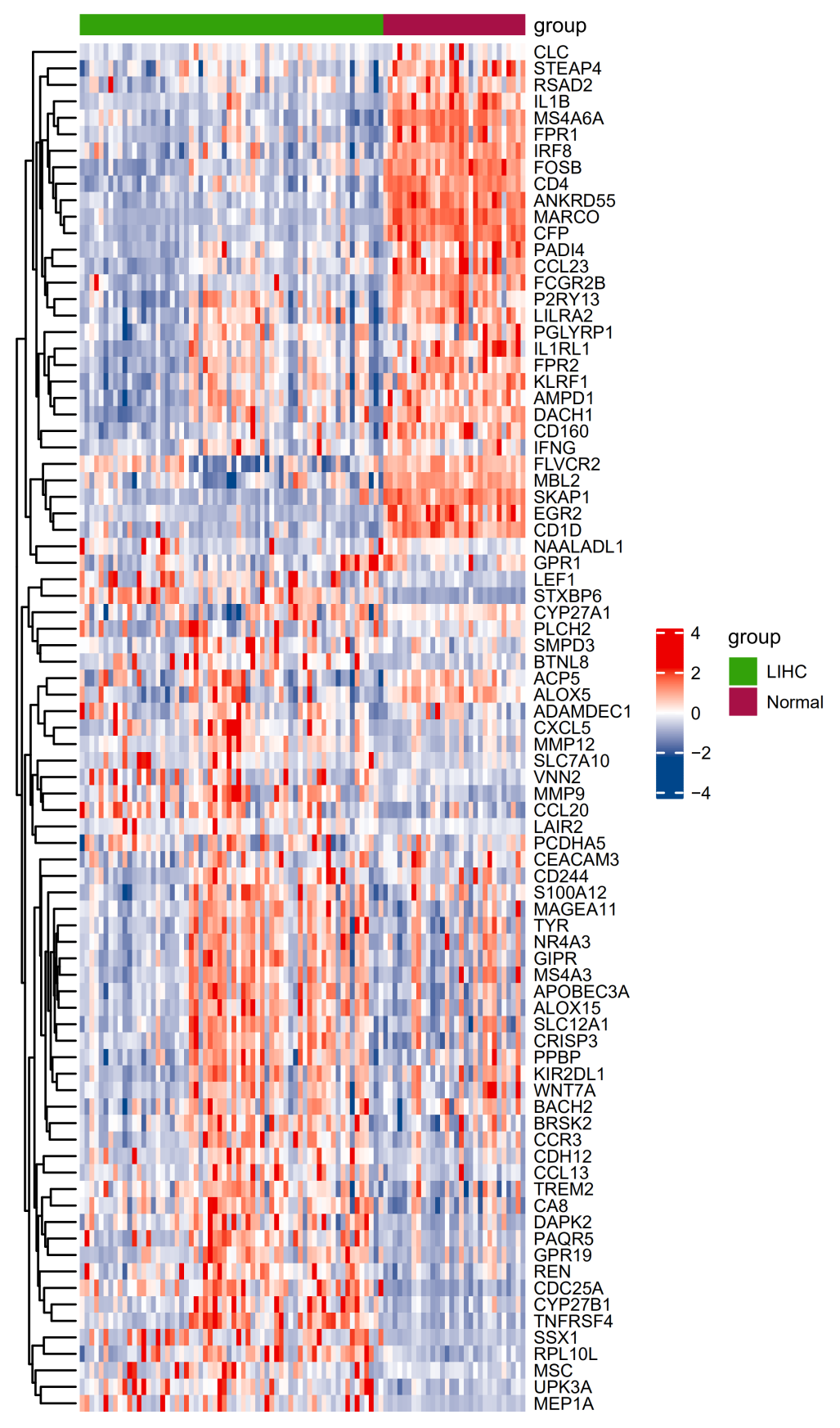


**Figure S1. Heat map of 89 DEGs related to immune infiltration in the data set GSE87630.**

Supplement: Supplementary file 4 — Additional file 4: Figure S1. Heat map of 89 DEGs related to immune infiltration in the data set GSE87630. [file 12885_2022_9587_MOESM4_ESM.docx]

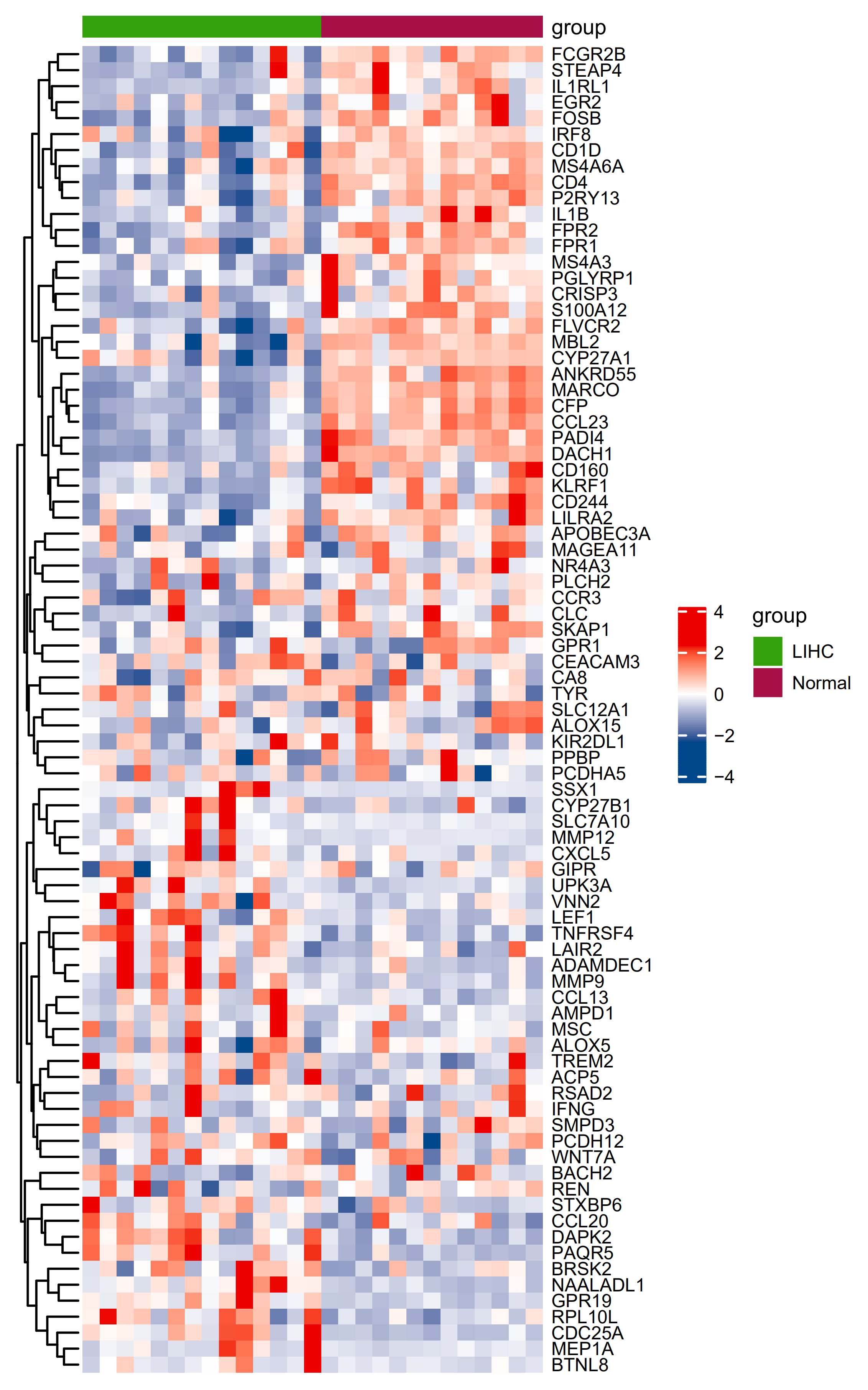


**Figure S2. Heat map of 89 DEGs related to immune infiltration in the data set GSE89377.**

Supplement: Supplementary file 5 — Additional file 5: Figure S2. Heat map of 89 DEGs related to immune infiltration in the data set GSE89377. [file 12885_2022_9587_MOESM5_ESM.docx]
