# Supplementary material for: Identification of immune infiltration-related genes as prognostic indicators for hepatocellular carcinoma
Source: BMC Cancer. 2022 May 5;22:496. doi: 10.1186/s12885-022-09587-0 (PMC9074323; doi:10.1186/s12885-022-09587-0)
Supplement: Supplementary file 6 — Additional file 6: Figure S3. KM survival curves of the 17 immune infiltration-related genes. (A) ORC1. (B) VNN2. (C) STEAP4. (D) SKA1. (E) BRSK2. (F) MSC. (G) CCR3. (H) IGHM. (I) CYP27A1. (J) DACH1. (K) TNFRSF4. (L) RPL10L. (M) CDC25A. (N) REN. (O) BACH2. (P) MMP9. (Q) CD4. [file 12885_2022_9587_MOESM6_ESM.docx]

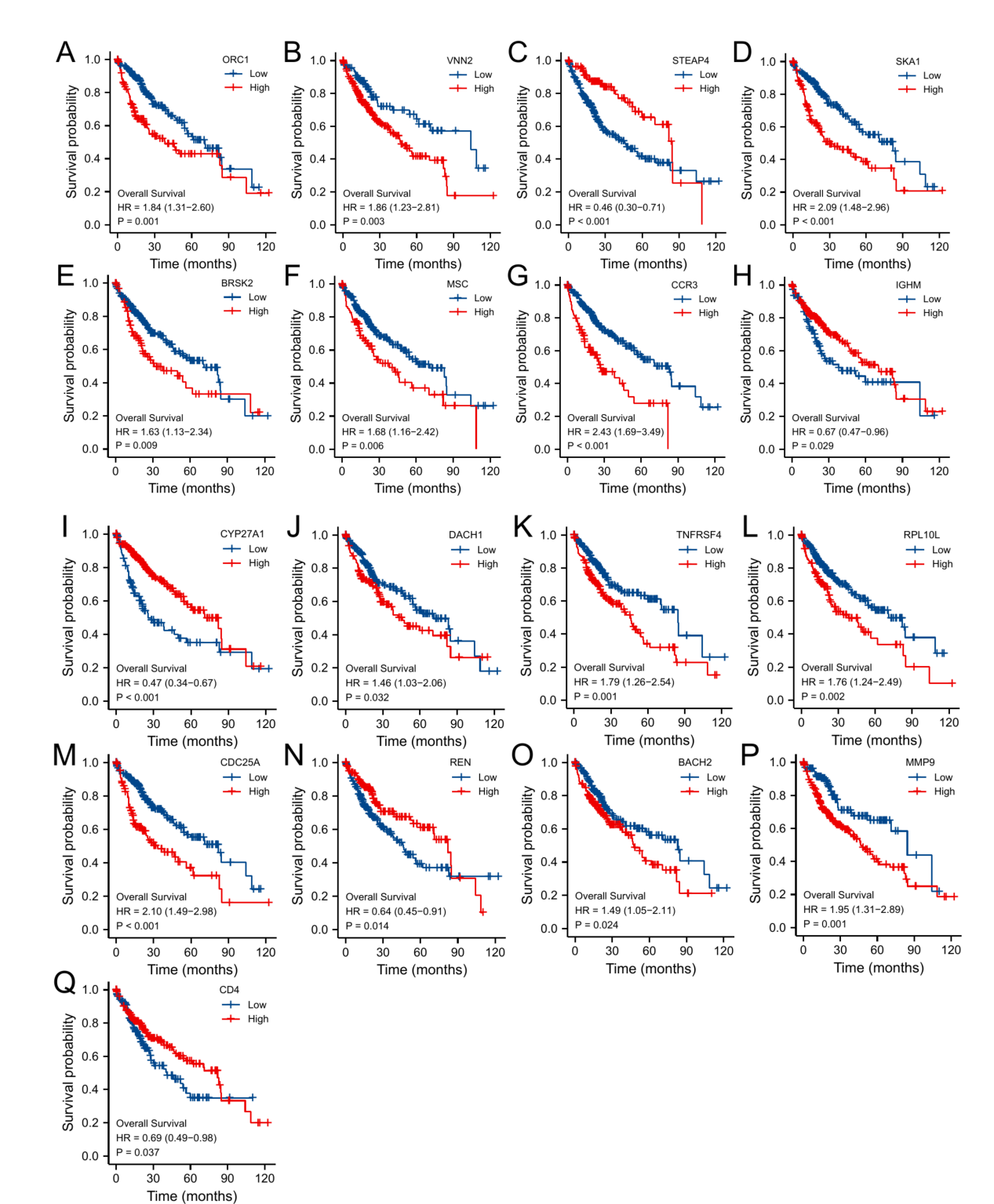


**Figure S3. KM survival curves of the 17 immune infiltration-related genes.** (A) ORC1. (B) VNN2. (C) STEAP4. (D) SKA1. (E) BRSK2. (F) MSC. (G) CCR3. (H) IGHM. (I) CYP27A1. (J) DACH1. (K) TNFRSF4. (L) RPL10L. (M) CDC25A. (N) REN. (O) BACH2. (P) MMP9. (Q) CD4.
